# Curative in vivo hematopoietic stem cell gene therapy of murine thalassemia using large regulatory elements

**DOI:** 10.1172/jci.insight.139538

**Published:** 2020-08-20

**Authors:** Hongjie Wang, Aphrodite Georgakopoulou, Chang Li, Zhinan Liu, Sucheol Gil, Ashvin Bashyam, Evangelia Yannaki, Achilles Anagnostopoulos, Amit Pande, Zsuzsanna Izsvák, Thalia Papayannopoulou, André Lieber

**Affiliations:** 1 Division of Medical Genetics, Department of Medicine, University of Washington, Seattle, Washington, USA.; 2 Hematology Department, Hematopoietic Stem Cell Transplantation Unit, George Papanicolaou Hospital, Thessaloniki, Greece.; 3 5AM Ventures, Boston, Massachusetts, USA.; 4 Max Delbrück Center for Molecular Medicine in the Helmholtz Association, Berlin, Germany.; 5 Division of Hematology, Department of Medicine, and; 6 Department of Pathology, University of Washington, Seattle, Washington, USA.

**Keywords:** Hematology, Therapeutics, Gene therapy, Hematopoietic stem cells, Mouse models

## Abstract

Recently, we demonstrated that hematopoietic stem/progenitor cell (HSPC) mobilization followed by intravenous injection of integrating, helper-dependent adenovirus HDAd5/35++ vectors resulted in efficient transduction of long-term repopulating cells and disease amelioration in mouse models after in vivo selection of transduced HSPCs. Acute innate toxicity associated with HDAd5/35++ injection was controlled by appropriate prophylaxis, making this approach feasible for clinical translation. Our ultimate goal is to use this technically simple in vivo HSPC transduction approach for gene therapy of thalassemia major or sickle cell disease. A cure of these diseases requires high expression levels of the therapeutic protein (γ- or β-globin), which is difficult to achieve with lentivirus vectors because of their genome size limitation not allowing larger regulatory elements to be accommodated. Here, we capitalized on the 35 kb insert capacity of HDAd5/35++ vectors to demonstrate that transcriptional regulatory regions of the β-globin locus with a total length of 29 kb can efficiently be transferred into HSPCs. The in vivo HSPC transduction resulted in stable γ-globin levels in erythroid cells that conferred a complete cure of murine thalassemia intermedia. Notably, this was achieved with a minimal in vivo HSPC selection regimen.

## Abstract 2

Employing large regulatory elements in the context of HDAd5/35++ vectors for in vivo transduction of HSPCs achieved gamma-globin levels in erythroid cells that cured murine thalassemia intermedia.

## Graphical abstract

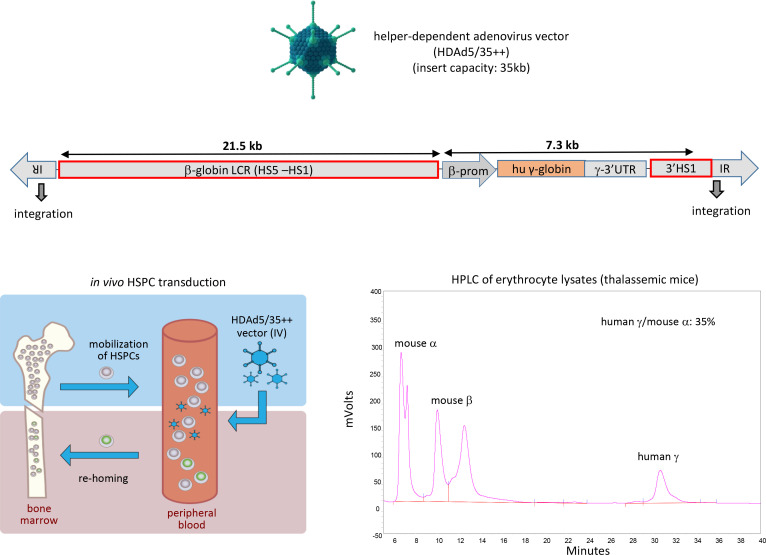

## Introduction

The approximately 100 kb human β-globin gene cluster lies in chromosome 11 and forms an erythroid-specific topologically associated domain (TAD) (1, 2). TADs are considered functional units of chromosome organization in which enhancers interact with other regulatory regions to control transcription. TAD border insulation is thought to restrict the search space of enhancers and promoters and to prevent unwanted regulatory contacts from being formed.

Due to their limited insert size capacity, currently used lentivirus and recombinant adeno-associated virus (rAAV) gene transfer vectors can accommodate only short enhancers/promoters, often resulting in suboptimal level of transgene expression, transgene silencing, and unintentional interactions with regulatory regions and genes surrounding the vector integration site. Hybrid transposon helper-dependent adenovirus (HDAd) integrating vectors offer a solution to the above problems as they have a large payload capacity (35 kb) and can carry TAD core elements (3).

We used capsid-modified HDAd5/35++ vectors for in vivo hematopoietic stem/progenitor cell (HSPC) gene therapy (4, 5). Our approach involves the mobilization of HSPCs from the bone marrow, and while they circulate at high numbers in the periphery, HDAd5/35++ vectors are injected intravenously. These vectors target CD46, a receptor that is expressed on primitive HSPCs (5). Transduced HSPCs return to the bone marrow, where they persist long term. Random integration is mediated by an activity-enhanced Sleeping Beauty transposase (SB100x) (6). Targeted integration can be achieved via homology-dependent DNA repair (7). We have recently shown that our approach results in an amelioration of murine thalassemia intermedia (8), the correction of murine hemophilia (9), and the reversion of spontaneous cancer (10). First data in nonhuman primates show that the in vivo HSPC gene therapy approach is safe when combined with glucocorticoid, IL-6, and IL-1β receptor antagonist pretreatment to suppress innate immune responses after intravenous HDAd5/35++ injection (11). The intravenous injection of HDAd5/35++ vectors did not result in transgene expression in tissues other than the mobilized HSPCs and PBMCs in CD46tg mice at day 3 after injection (5, 8). This was recently confirmed in nonhuman primates (unpublished data). A potential explanation for this tropism is that CD46 receptor density and accessibility are not sufficiently high in nonhematopoietic tissues to allow for efficient viral transduction (5, 12).

In a previous study with HDAd5/35++ vectors, we used a 4.3 kb hypersensitivity site 1–4 (HS1-HS4) mini–locus control region (β-globin LCR) in combination with a 0.66 kb β-globin promoter to drive human γ-globin expression after in vivo HSPC transduction (8, 13). In Hbb^th3^ CD46^+/+^ thalassemic mice, we achieved stable (8+ months) γ-globin marking in nearly 100% of peripheral blood erythrocytes and near complete phenotypic correction (8). However, the level of γ-globin expression was only 10%–15% of that of adult mouse α-globin with an average integrated vector copy number (VCN) of 2 copies per cell, thus rendering the clinical translation of the approach to thalassemia major or sickle cell disease (SCD) particularly challenging. Here, we aimed to exploit the large capacity of HDAd5/35++ vectors by incorporating β-globin TAD core elements comprising a γ-globin expression cassette with a length of 29 kb to achieve complete phenotypic correction.

In this context, we also set out to demonstrate that the SB100x system can mediate the efficient integration of a 32.4 kb transposon. From studies with plasmid-based Sleeping Beauty (SB) systems, it was thought that the SB integration activity negatively correlated with the length of the transposon (14, 15). Considering this, the first SB-based HDAd vectors the Kay and Ehrhardt groups developed carried relatively small (4–6 kb) transposons (16, 17). Recently, using HDAd5/35++ vectors, we demonstrated efficient SB100x-mediated integration of 10.8 kb (9) and 11.8 kb (8, 13) transposons in HSPCs after ex vivo or in vivo HSPC transduction. Here, we provide proof that the HDAd5/35++-based SB100x vector system can integrate a 32.4 kb transposon.

Overall, our in vivo studies in normal and thalassemic mice as well as in vitro studies with human CD34^+^ cells indicate that our long-LCR containing HDAd5/35++ vector could be an efficient therapeutic tool for the treatment of hemoglobinopathies.

## Results

### HDAd5/35++ vector containing a long β-globin LCR.

**Figure 1:**
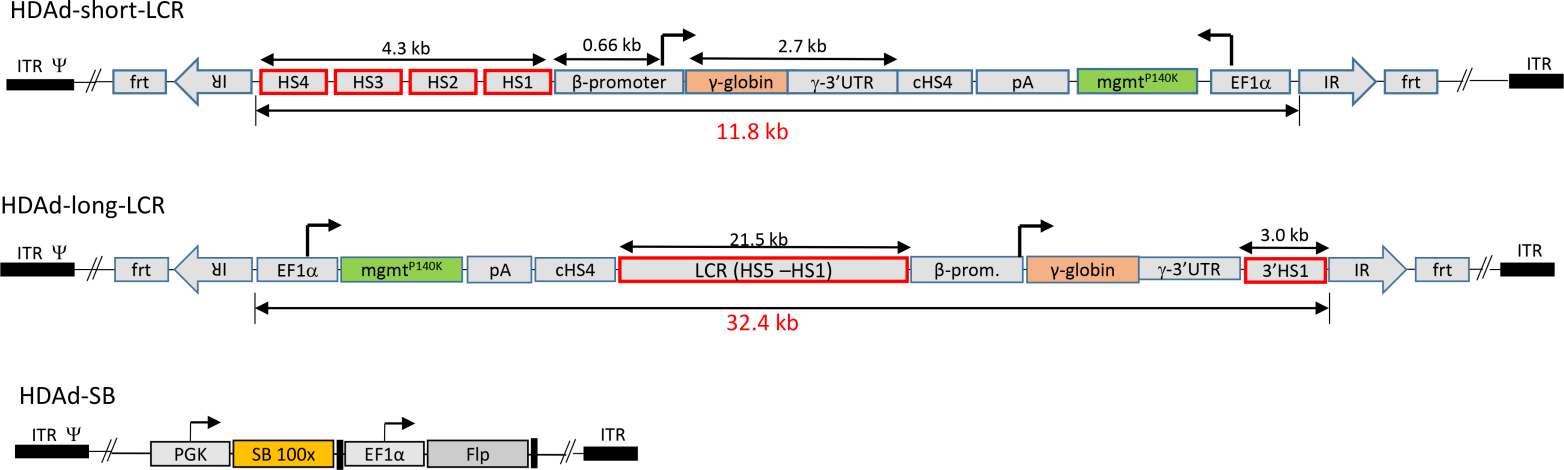
Vector structures. *HDAd-short-LCR*: This vector contains a 4.3 kb mini-LCR consisting of the core regions of DNase hypersensitivity sites (HS) 1 to 4 and a 0.66 kb β-globin promoter. The length of the transposon is 11.8 kb. *HDAd-long-LCR*: The γ-globin gene is under the control of a 21.5 kb β-globin LCR (chr11: 5292319-5270789), a 1.6 kb β-globin promoter (chr11: 5228631-5227023), and a 3′HS1 region (chr11: 5206867-5203839) also derived from the β-globin locus. For RNA stabilization in erythroid cells, a γ-globin gene UTR was linked to the 3′ end of the γ-globin gene. The vector also contains an expression cassette for mgmt^P140K^ allowing for in vivo selection of transduced HSPCs and HSPC progeny. The γ-globin and mgmt expression cassettes are separated by a chicken globin HS4 insulator. The 32.4 kb LCR-γ-globin/mgmt transposon is flanked by inverted repeats (*IR*s) that are recognized by SB100x and by *frt* sites that allow for the circularization of the transposon by Flpe recombinase. *HDAd-SB:* The second vector required for integration contains the expression cassettes for the activity-enhanced SB100x transposase and the Flpe recombinase. ITR, inverted terminal repeat; frt, flippase recognition target; pA, polyadenylation signal; EF1α, elongation factor 1α.

We used the above-described HDAd5/35++ vector (Figure 1, “HDAd-short-LCR”) (8). Furthermore, we generated a HDAd5/35++ vector that contained the following elements to maximize γ-globin gene expression: (a) a 21.5 kb LCR including the full-length HS5 to HS1 regions, (b) a 1.6 kb β-globin promoter, (c) a γ-globin 3′UTR to stabilize γ-globin mRNA, and (d) a 3′HS1 region. The vector was named HDAd-long-LCR (Figure 1). To mediate integration, the LCR vectors are used in combination with a SB100x/Flpe expressing HDAd vector (Figure 1). The transposon vectors (HDAd-short-LCR and HDAd-long-LCR) contain inverted/direct repeat (IR/DR) motifs, which are recognized by the SB100x transposase and *frt* sites that allow for circularization of the transgene cassette in the presence of Flpe recombinase. Both HDAd-short-LCR and HDAd-long-LCR also carried the gene for a mutant O6-methylguanine-DNA methyltransferase (mgmt^P140K^) under control of the ubiquitously active human EF1α promoter to allow for selection of stably transduced cells by low-dose O^6^-benzylguanine/carmustine (O^6^BG/BCNU) treatment (18, 19).

### Ex vivo HSPC transduction/transplantation study.

While in humans, CD46 is expressed on all nucleated cells, the corresponding orthologue in mice is present only in the testes. As a model for our in vivo transduction studies with intravenously injected HDAd5/35++ vectors, we used transgenic mice that contained the complete human CD46 locus and therefore expressed hCD46 in a pattern and at a level similar to humans (CD46tg mice) (20). Because, a priori, it was not known whether SB100x can mediate the integration of the 32.4 kb transposon, we performed ex vivo HSPC transduction studies, in a setting where we could control the HSPC transduction efficacy. CD46tg mouse bone marrow lineage–negative (Lin^–^) cells, a cell fraction enriched for HSPCs, were transduced ex vivo with HDAd-long-LCR + HDAd-SB (Supplemental Figure 1A; supplemental material available online with this article; https://doi.org/10.1172/jci.insight.139538DS1). Ex vivo–transduced cells were then transplanted into lethally irradiated C57BL/6 mice. Engraftment rates at week 4 were more than 95% based on CD46^+^ PBMCs. One month after transplantation, mice were subjected to 4 rounds of O^6^BG/BCNU treatment to selectively expand progenitors with integrated γ-globin/mgmt transgenes. With each round of in vivo selection, the percentage of γ-globin–positive peripheral red blood cells (RBCs) increased, reaching more than 95% at the end of the study (Supplemental Figure 1B). At week 20, animals were sacrificed. To demonstrate that γ-globin expression originated from SB100x integrated transgenes, we performed an inverse PCR (iPCR) analysis on genomic DNA from bone marrow mononuclear cells (MNCs) (Supplemental Figure 1C). Supplemental Figure 1D shows 3 representative PCR products and the localization of the integration sites on chromosomes 4, 15, and X. Sequencing of the products demonstrated vector/chromosome junctions typical for SB100x-mediated integration, including the TA dinucleotides at the vector IR/DR chromosome junctions (Supplemental Figure 1E).

### In vivo HSPC transduction in CD46tg mice with HDAd5/35++ vectors containing short versus long LCRs.

**Figure 2:**
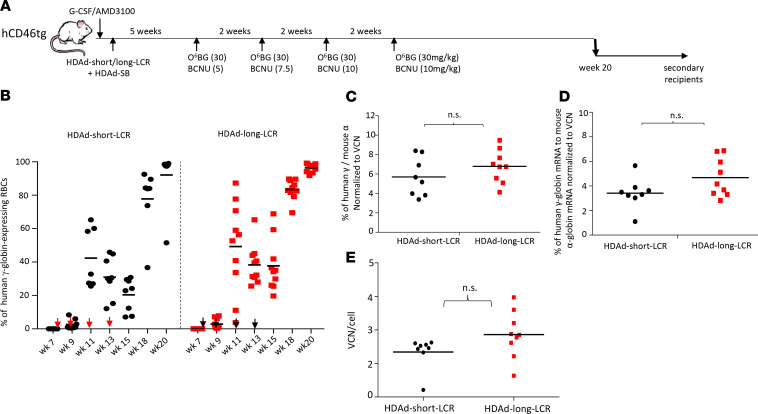
In vivo HSPC transduction with HDAd-long-LCR containing the 32.4 kb transposon and HDAd-short-LCR containing an 11.8 kb transposon. (**A**) Treatment regimen: hCD46tg mice were mobilized and IV injected with either HDAd-short-LCR + HDAd-SB or HDAd-long-LCR +HDAd-SB (2 times each 4 × 10^10^ viral particles (vp) of a 1:1 mixture of both viruses). Five weeks later, O^6^BG/BCNU treatment was started. With each cycle, the BCNU concentration was increased from 5 mg/kg, to 7.5 mg/kg, and to 10 mg/kg. The O^6^BG concentration was 30 mg/kg in all 4 treatments. Mice were followed until week 20, when animals were sacrificed for analysis. Bone marrow Lin^–^ cells were used for transplantation into secondary recipients. Secondary recipients were then followed for 16 weeks. (**B**) Percentage of human γ-globin–positive cells in peripheral red blood cells (RBCs) measured by flow cytometry. Each symbol is an individual animal. In mice that were mock transduced, fewer than 0.1% of cells were γ-globin–positive. The arrows indicate O^6^BG/BCNU treatment. (**C**) Levels of γ-globin protein chain measured by HPLC in RBCs at week 20 after in vivo HSPC transduction. Shown are the percentages of human γ-globin to mouse α-globin protein chains. (**D**) Levels of γ-globin mRNA measured by quantitative reverse transcription (qRT-PCR) in total blood at week 20 after in vivo HSPC transduction. Shown are the percentages of human γ-globin mRNA to mouse α-globin mRNA. (**E**) VCN per cell in bone marrow MNCs, harvested at week 20 after in vivo HSPC transduction. The difference between the 2 groups is not significant. Statistical analyses were performed using 2-way ANOVA.

**Figure 3:**
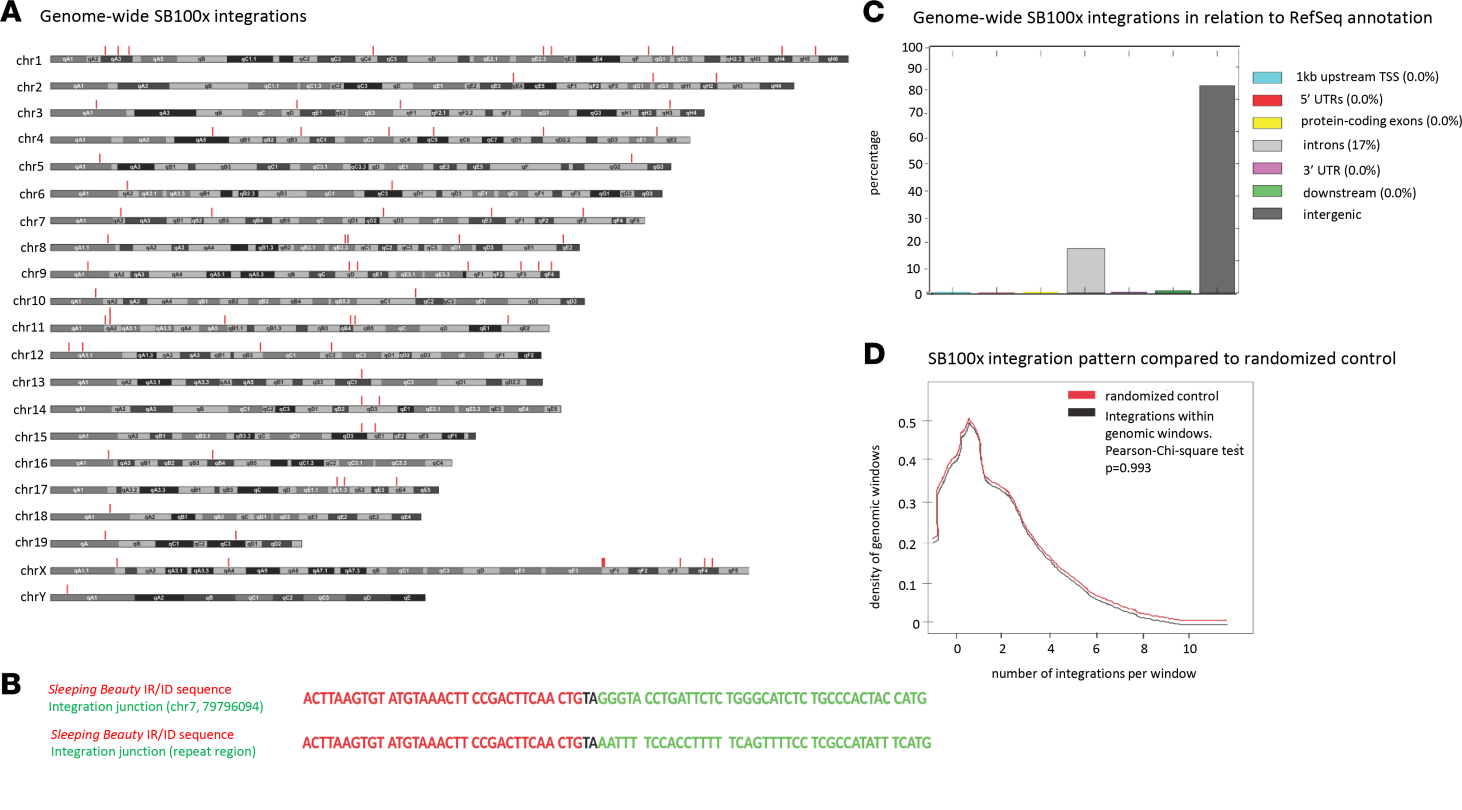
Analysis of vector integration sites in HSPCs by LAM-PCR/next-generation sequencing. Genomic DNA was isolated from bone marrow cells harvested at week 20 after in vivo transduction with HDAd-long-LCR + HDAd-SB. (**A**) Chromosomal distribution of integration sites. The integration sites are marked by vertical red lines. (**B**) Examples for junction sequences. IR/DR sequences are in red. The chromosomal sequence is in green. The TA dinucleotides used by SB100x at the junction of the IR and chromosomal DNA are highlighted. (**C**) Integration sites were mapped to the mouse genome, and their location with respect to genes was analyzed. Shown is the percentage of integration events that occurred 1 kb upstream transcription start sites, 3′UTR of exons, protein coding sequences, introns, 3′UTRs, 1 kb downstream from 3′UTR, and intergenic. (**D**) Integration pattern in mouse genomic windows. The number of integrations overlapping with continuous genomic windows and randomized mouse genomic windows and size was compared. This shows that the pattern of integration is similar in continuous and random windows. Maximum number of integrations in any given window was not more than 3, with 1 integration per window having the higher incidence.

We next performed a side-by-side comparison of HDAd-short-LCR and HDAd-long-LCR. CD46tg mice were mobilized with G-CSF/AMD3100, intravenously injected with the vectors, and, 5 weeks later, subjected to in vivo selection (Figure 2A). The percentage of γ-globin–positive RBCs increased with each round of in vivo selection, reaching more than 95% for both vectors at week 20 (Figure 2B). HPLC performed on RBC lysates from week 20 samples did not show significant differences in percentages of γ-globin/adult mouse α-globin between the vectors (Figure 2C). This was also reflected at the mRNA level (Figure 2D). The VCN in bone marrow MNCs, measured at week 20 by quantitative PCR (qPCR), was approximately 2.5 copies per cell (Figure 2E) and not significantly different between the vectors. This indicated that the integration of the “long” 32.4 kb transposon was as efficient as the integration of the “short” 11.8 kb transposon. To demonstrate SB100x-mediated integration of the 32.4 kb transposon after in vivo HSPC transduction, we subjected bone marrow cells harvested at week 20 to a genome-wide integration site analysis. In this assay, a linear amplification-mediated PCR (LAM-PCR) strategy is followed by sequencing of integration junctions (Supplemental Figure 2). The distribution of integration sites over the mouse genome is shown in Figure 3A. The integrated transgene cassette was precisely processed, and the identified IR/DR chromosome junctions contained TA dinucleotides (Figure 3B). The vast majority of integrations were within intergenic and intronic regions at a frequency of 83% and 17%, respectively (Figure 3C). The integration was random without preferential integration in any given window of the whole mouse genome (Figure 3D). No integration within or near a proto-oncogene was found. This SB100x-mediated integration pattern is in agreement with previous studies (5, 17, 19, 21–23).

**Figure 4:**
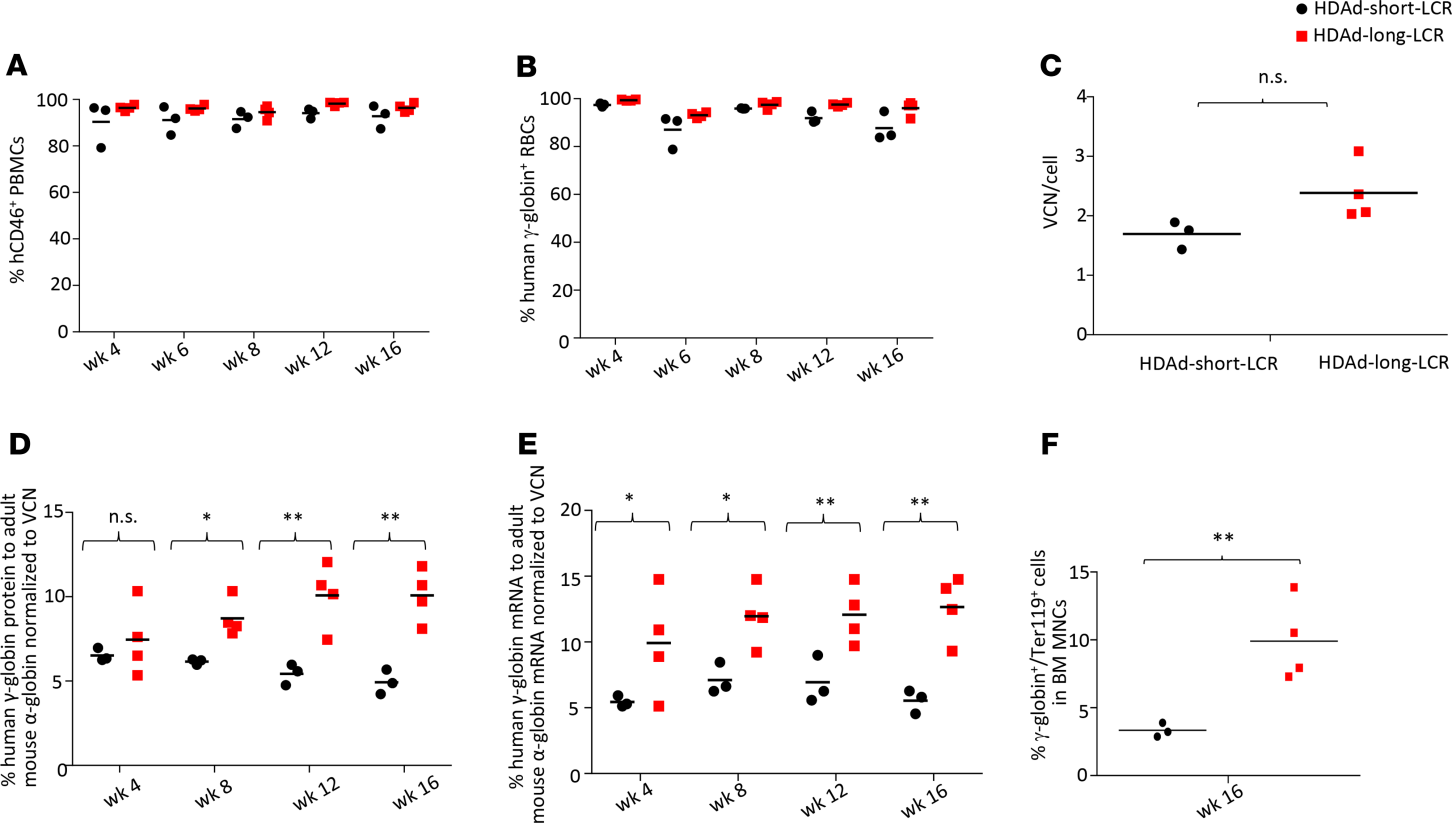
Analysis of secondary recipients. Bone marrow Lin^–^ cells harvested at week 20 from in vivo–transduced CD46tg mice were transplanted into lethally irradiated C57BL/6 mice. Secondary recipients were followed for 16 weeks. (**A**) Engraftment rates based on the percentage of CD46^+^ PBMCs at weeks 4, 8, 12, and 16 after transplantation. The differences between the 2 groups were not significant. (**B**) Percentage of γ-globin–expressing peripheral blood RBCs measured by flow cytometry. The differences between the 2 groups are not significant. (**C**) VCN per cell in bone marrow MNCs harvested at week 20 after in vivo HSPC transduction. The difference between the 2 groups is not significant. (**D**) Analysis of human γ-globin chains by HPLC in RBCs of secondary recipients. Shown is the percentage of human γ-globin to adult mouse α-globin. (**E**) Levels of γ-globin mRNA in total blood cells relative to mouse α-globin mRNA. (**F**) Percentage of γ-globin–expressing erythroid (Ter119^+^ cells) in all bone marrow MNCs. *P < 0.05; **P < 0.001. Statistical analyses were performed using 2-way ANOVA.

To demonstrate that in vivo transduction occurred in long-term repopulating HSPCs, we transplanted bone marrow Lin^–^ cells, harvested at week 20 after in vivo HSPC transduction with HDAd-short-LCR and HDAd-long-LCR, into lethally irradiated C57BL/6 mice (without the hCD46 transgene). The ability of transplanted cells to drive the multilineage reconstitution in secondary recipients was assessed over 16 weeks. Engraftment rates based on CD46 expression in PBMCs were approximately 95% and remained stable (Figure 4A). γ-Globin marking of RBCs measured by flow cytometry was in the range of 90% to 95% and stable (Figure 4B). There was no significant difference between the 2 vectors in the percentage of γ-globin–positive RBCs. The average integrated VCN also did not differ significantly between the 2 vectors, indicating that integration of both transposons in long-term repopulating cells was equally efficient (Figure 4C). Interestingly, the percentage of γ-globin to mouse adult globin chains increased over time for the HDAd-long-LCR vector, reaching 20%–25% of mouse α-globin (Figure 4, D and E). In contrast, the percentage of γ-globin/mouse α-globin in secondary recipients of HDAd-short-LCR transduced bone marrow cells did not increase. The percentage of γ-globin–expressing erythroid cells was significantly higher for HDAd-long-LCR (Figure 4F). No effect of high-level globin expression on the cellular composition of the bone marrow or hematological parameters in the peripheral blood was observed (Supplemental Figure 3).

### HDAd-short-LCR versus HDAd-long-LCR in vivo HSPC transduction studies in a mouse model of thalassemia intermedia — γ-globin levels.

**Figure 5:**
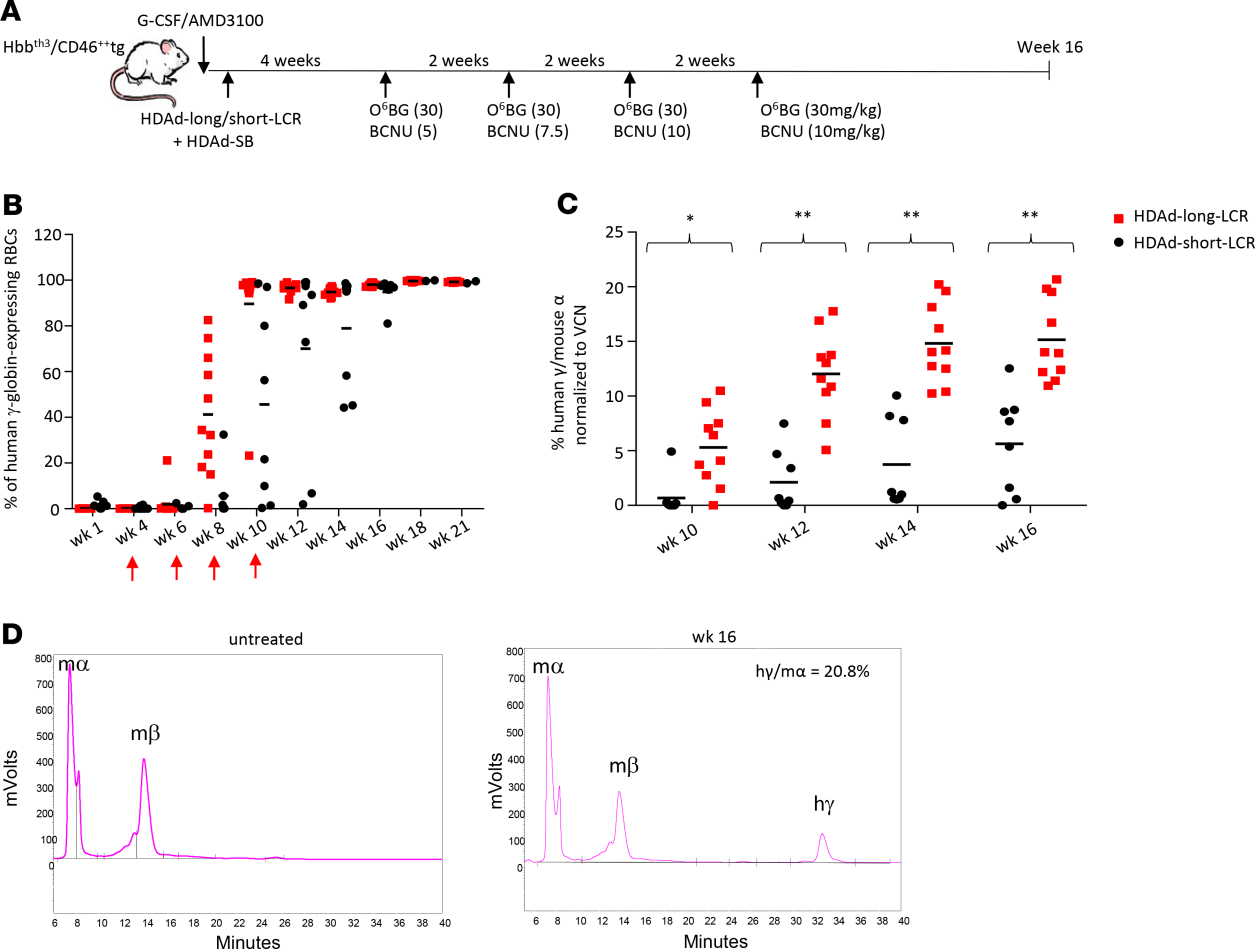
Human γ-globin expression after in vivo HSPC gene therapy of Hbb^th3^ CD46^+/+^ mice with HDAd-short-LCR and HDAd-long-LCR. (**A**) Treatment regimen: In contrast to the study shown in Figure 2, this study was done with thalassemic Hbb^th3^ CD46 mice. (**B**) Percentage of human γ-globin–positive cells in peripheral RBCs measured by flow cytometry. Each symbol is an individual animal. The arrows indicate O^6^BG/BCNU treatment. (**C**) γ-Globin protein chain levels measured by HPLC in RBCs at weeks 10 to 16 after in vivo HSPC transduction. Shown are the percentages of human γ-globin to mouse α-globin protein chains. (**D**) Representative chromatograms of an untreated Hbb^th3^ CD46^+/+^ mouse (left panel) and a mouse at week 16 after treatment. Mouse α- and β-chains as well the added human γ-globin are indicated. Notably, 2 independent studies were performed with Hbb^th3^ CD46^+/+^ mice. First study: *N* = 6 for HDAd-long-LCR and *N* = 2 for HDAd-short-LCR followed for 21 weeks. Second study: *N* = 4 for HDAd-long-LCR and *N* = 5 for HDAd-short-LCR followed for 16 weeks. Figure 5B shows the combined data until week 21. All remaining figures show the combined data until week 16. Statistical analyses were performed using 2-way ANOVA. *P < 0.05; **P < 0.0001.

For these studies we bred (over 4 rounds) (CD46^+/+^) mice with B6.D2-*Hbb^d3th^*/BrkJ (Hbb^th3^) mice heterozygous for the mouse Hbb-beta1 and -beta2 gene deletion (24). Resulting Hbb^th3^ CD46^+/+^ mice have the typical phenotype of thalassemia intermedia (8). Hbb^th3^ CD46^+/+^ mice were mobilized, intravenously injected with HDAd-long-LCR and HDAd-short-LCR vector systems, and 4 weeks later subjected to in vivo selection (Figure 5A). Importantly, γ-globin marking in peripheral RBCs was on average 40% already after the second cycle of in vivo selection, reached more than 90% in 9 out of 10 mice after the third cycle, and plateaued near 100% in all mice at week 12 after in vivo transduction with HDAd-long-LCR (Figure 5B). In contrast, for mice transduced with HDAd-short-LCR, it required 4 in vivo selection cycles to reach 100% γ-globin marking in RBCs in 2 out of 7 mice, and 100% marking was achieved only at week 16 posttransduction. At the 100% marking rate, the percentage of human γ-globin versus adult mouse α-globin chains (measured by HPLC) increased over time for both vectors (most likely due to the disease background), reaching an average of 22% (max: 35%) and 11% (max: 19%) by week 16 after in vivo transduction with HDAd-long-LCR and HDAd-short-LCR, respectively (Figure 5, C and D). Similar to what we observed in CD46tg mice, analysis of bone marrow MNCs showed comparable VCNs for both vectors and higher globin expression levels in erythroid cells for HDAd-long-LCR (Supplemental Figure 4).

In summary, these data demonstrate the superiority of HDAd-long-LCR over HDAd-short-LCR by (a) requiring less intense in vivo selection to reach 100% marking and (b) achieving γ-globin levels in RBCs, that, in theory, should be curative in patients with SCD and thalassemia major.

### Correction of hematological parameters.

**Figure 6:**
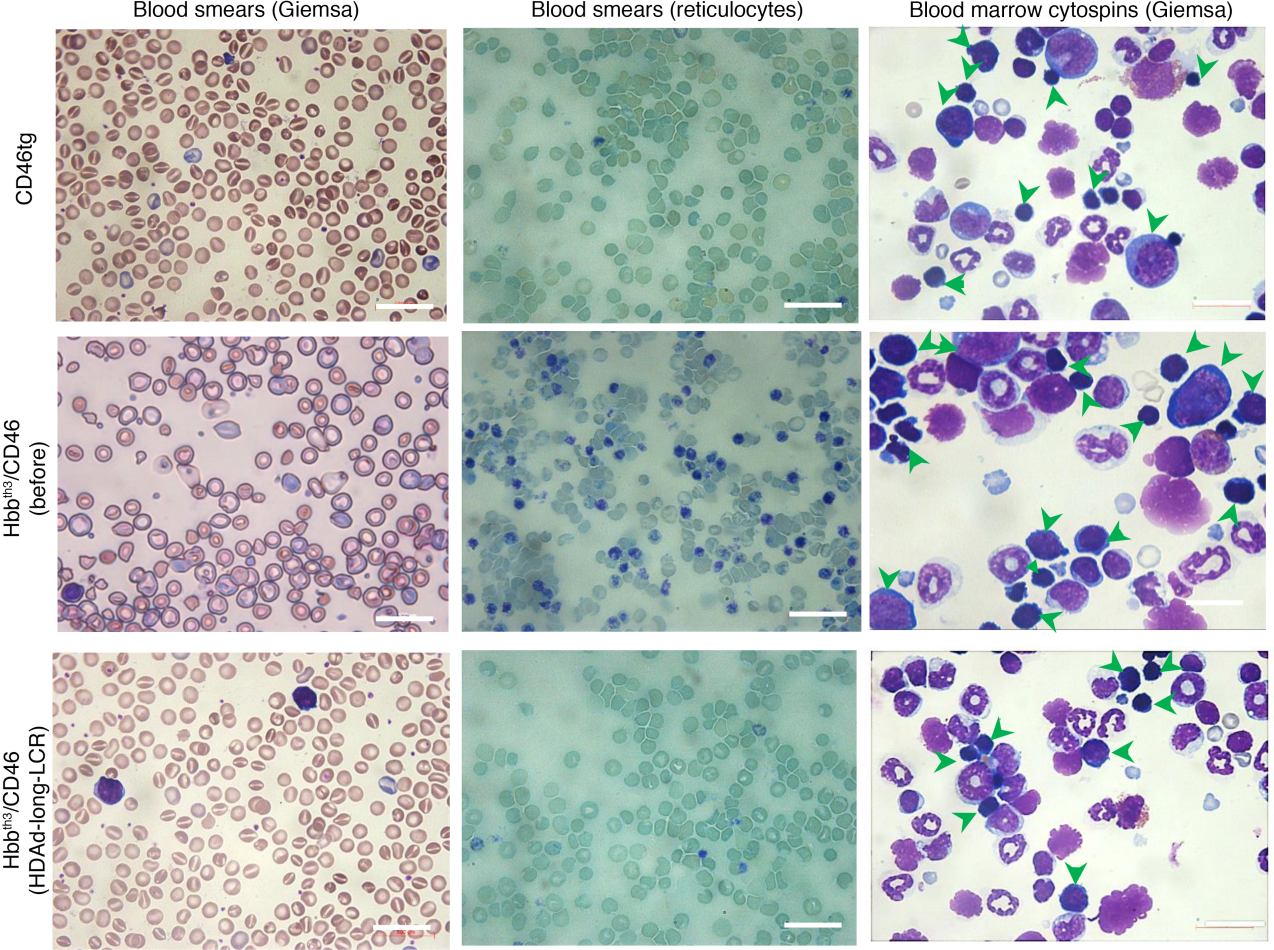
Phenotypic correction (week 16). Left panels: Blood smears stained with Giemsa/May-Grünwald stain (5 minutes). Middle panels: Blood smears stained with Brilliant cresyl blue for reticulocytes. Remnants of nuclei and cytoplasm in reticulocytes appear as purple staining. Right panels: Bone marrow cytospins stained with Giemsa/May-Grünwald stain (15 minutes). Upper panel: Normal bone marrow cellular distribution – erythroid lineage is represented by all stages of erythrocyte differentiation. Middle panel: Predominance of erythroid lineage over white cell lineage – erythroid lineage consists mainly of proerythroblasts and basophilic erythroblasts. Bottom panel: Normal bone marrow cellular distribution – erythroid lineage is mainly represented by maturing polychromatic and orthochromatic erythroblasts. Scale bars: 25 μm.

**Figure 7:**
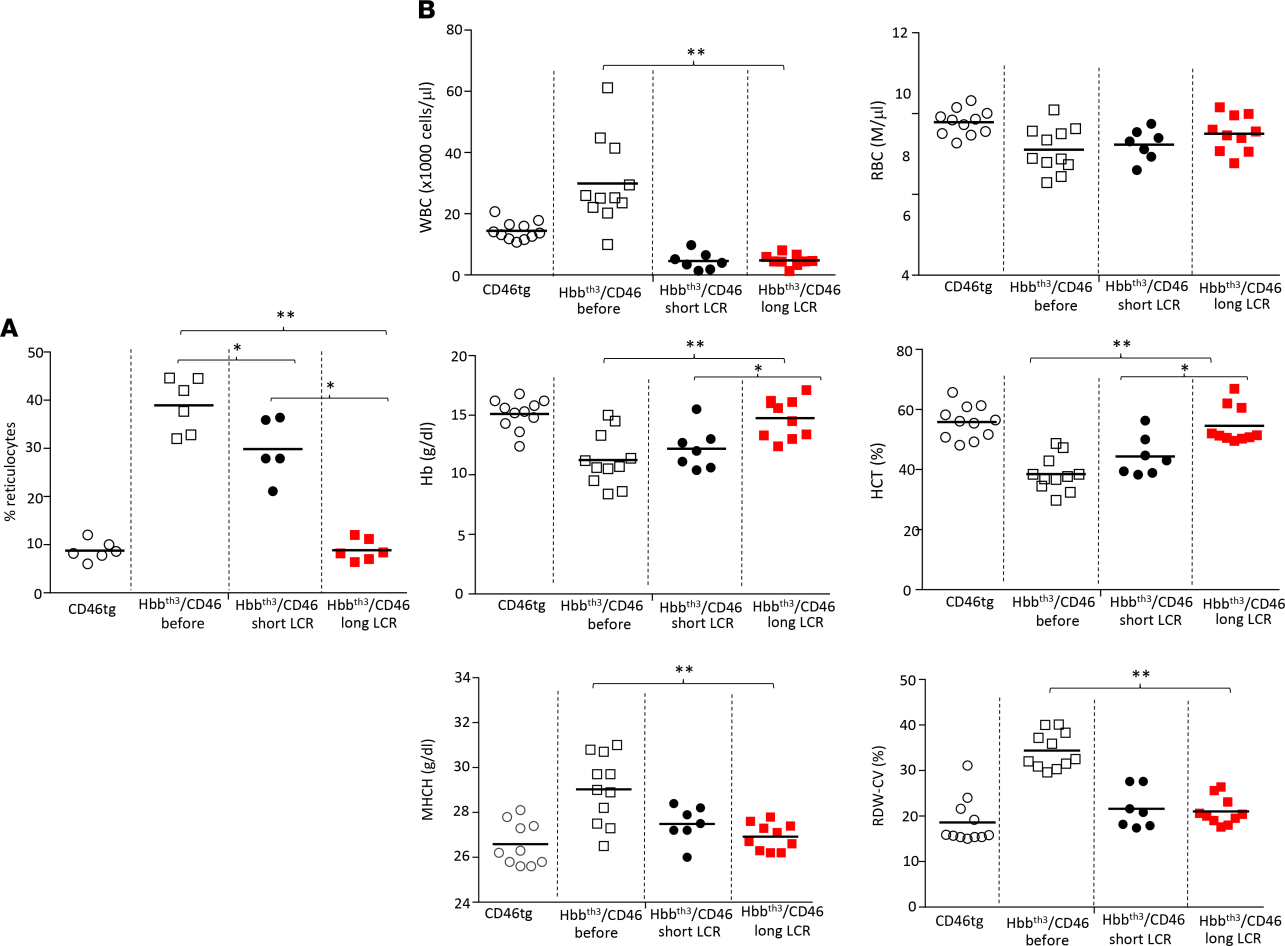
Hematological parameters before and after in vivo HSPC gene therapy of Hbb^th3^ CD46^+/+^ mice (week 16). (**A**) Reticulocyte counts. (**B**) Hematological parameters. Statistical analyses were performed using 2-way ANOVA. *P < 0.05; **P < 0.0001.

At week 16 after treatment, mice were sacrificed. Indicative of the reversal of the thalassemic phenotype in peripheral blood smears of the treated Hbb^th3^ CD46^+/+^ mice, the hypochromic, highly fragmented, and anisopoikilocytic baseline RBCs were replaced by nearly normochromic, well-shaped RBCs (Figure 6). The level of reticulocytes in peripheral blood was comparable to normal CD46tg mice (Figure 6; see also Figure 7A). In bone marrow cytospins, in contrast to the blockade of erythroid lineage maturation in bone marrow of Hbb^th3^ CD46^+/+^ mice, represented by the prevalence of basophilic erythroblasts, in cytospins from control and treated Hbb^th3^ CD46^+/+^ mice, maturing polychromatic and orthochromatic erythroblasts predominated (Figure 6). The cellular bone marrow composition of CD46-transgenic and treated Hbb^th3^ CD46^+/+^ mice at week 16 after in vivo transduction was not significantly different (Supplemental Figure 5).

Hematological parameters at week 16 after in vivo transduction were significantly improved compared with pretreatment parameters for both vectors (Figure 7, A and B). WBCs, RBCs, mean corpuscular hemoglobin concentration (MCHC), mean corpuscular volume (MCV), and red blood cell distribution width (RDW-CV) were indistinguishable from the CD46tg controls (Figure 7B). However, there were significant differences in favor of animals treated with HDAd-long-LCR vector versus HDAd-short-LCR; specifically, the percentage of reticulocytes in peripheral blood was 40.9% versus 26.8% versus 9.2% for nontreated, HDAd-short-LCR–, and HDAd-long-LCR–treated Hbb^th3^ CD46^+/+^ mice, respectively (Figure 7A). Furthermore, hemoglobin levels and hematocrit were higher for the HDAd-long-LCR–treated group.

### Correction of extramedullary hematopoiesis and hemosiderosis.

**Figure 8:**
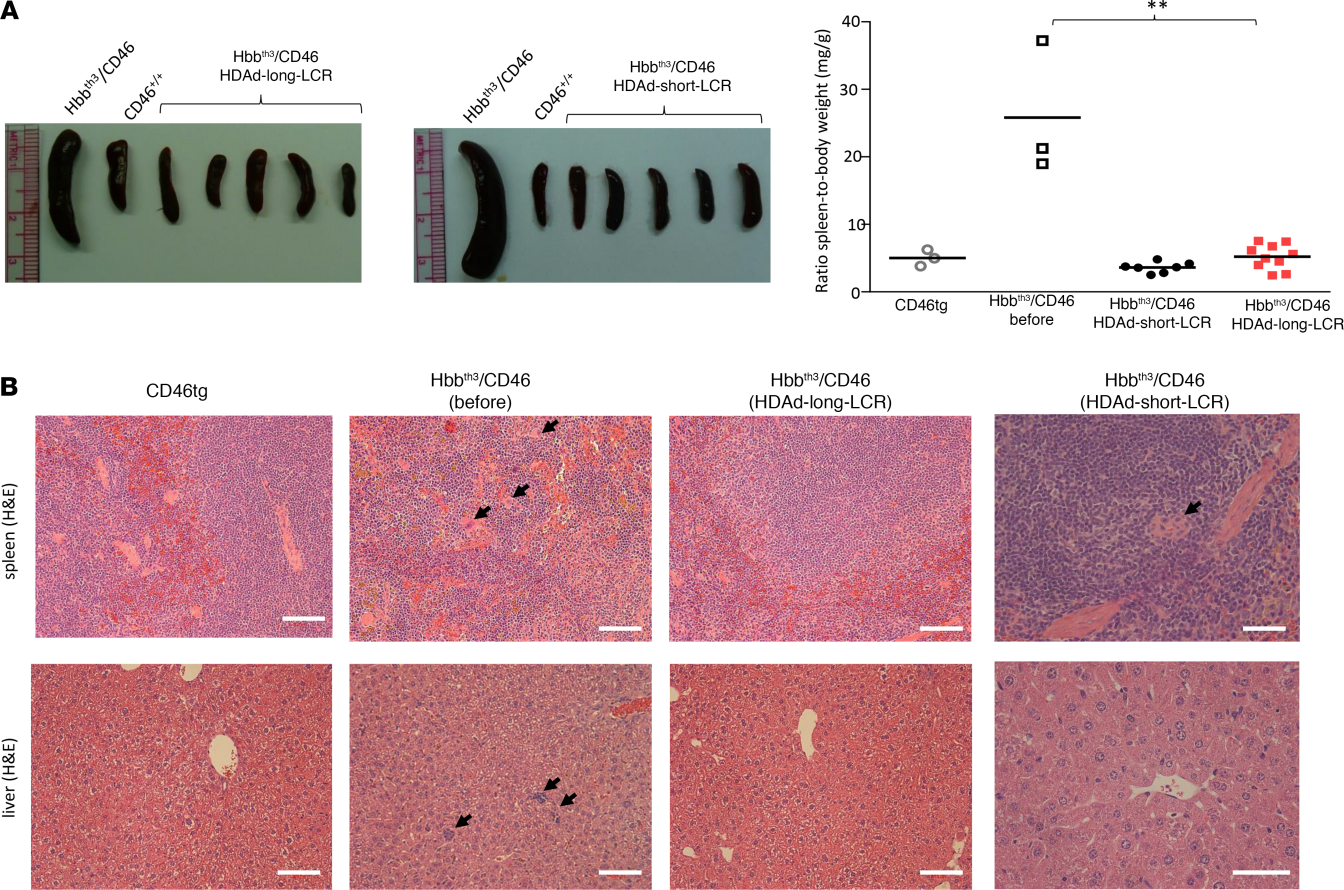
Phenotypic correction of extramedullary hematopoiesis in spleen and liver. (**A**) Spleen size at sacrifice (week 16). Left panel: representative spleen images. Right panel: summary. Each symbol represents an individual animal. Statistical analysis was performed using 1-way ANOVA. **P < 0.0001. The difference between the 2 vectors is not significant. (**B**) Extramedullary hemopoiesis by H&E staining in liver and spleen sections. Clusters of erythroblasts in the liver and megakaryocytes in the spleen of Hbb^th3^ CD46^+/+^ mice are indicated by black arrows. Scale bars: 20 μm. Representative images are shown.

**Figure 9:**
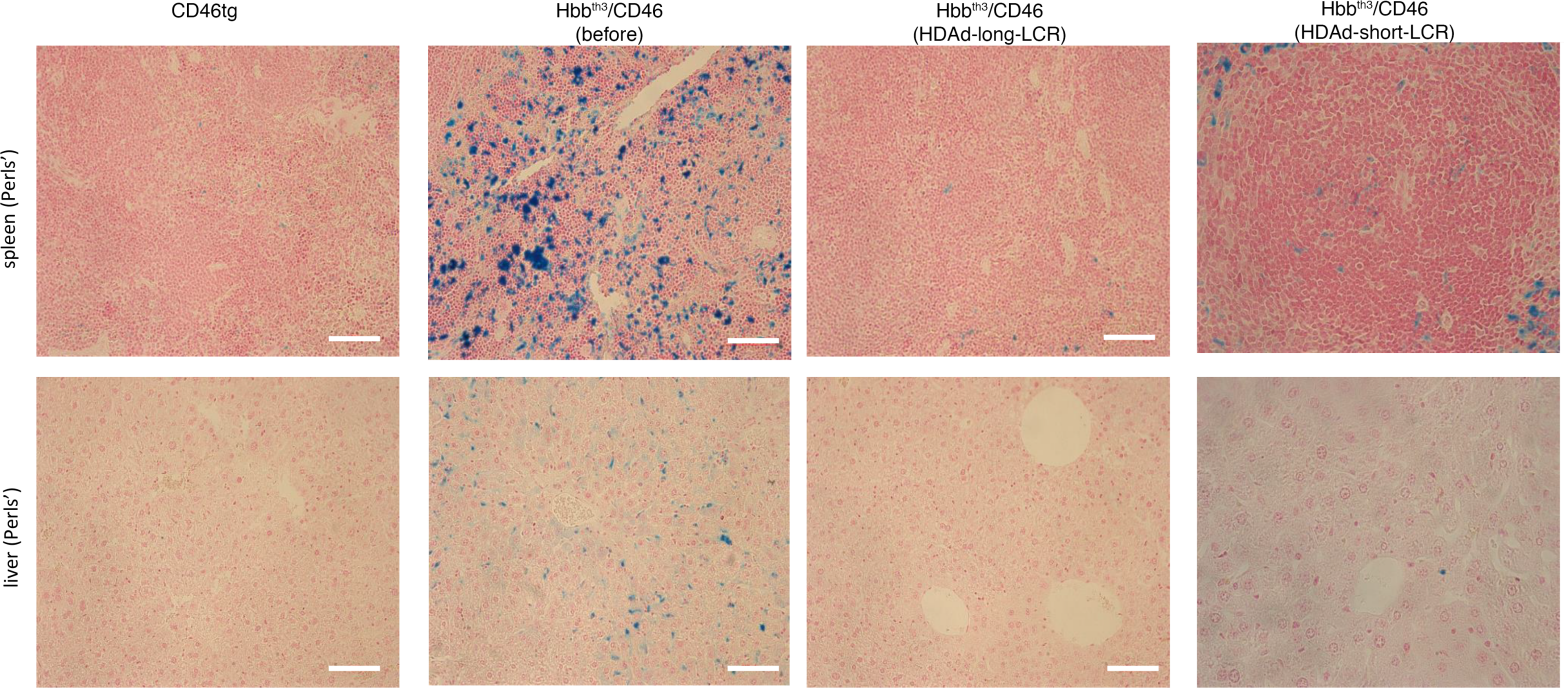
Phenotypic correction of hemosiderosis in spleen and liver (week 16). Iron deposition is shown by Perls’ staining as cytoplasmic blue pigments of hemosiderin in spleen and liver sections. Scale bars: 20 μm. Representative sections are shown.

Spleen size, a measurable characteristic of compensatory hemopoiesis, was reduced to normal in animals treated with both vectors, whereby there was no significant difference between HDAd-long-LCR and HDAd-short-LCR (Figure 8A). In contrast to Hbb^th3^ CD46^+/+^ mice, no foci of extramedullary erythropoiesis were observed on spleen and liver sections after treatment with HDAd-long-LCR, and only limited extramedullary erythropoiesis was detected in the HDAd-short-LCR–treated mice (Figure 8B). Intense hemosiderosis in spleen and liver was prominent in the untreated Hbb^th3^ CD46^+/+^ mice (Figure 9, second panel). Signals after Perls’ staining of the tissues were comparably low for CD46tg (Figure 9, first panel) and HDAd-long-LCR–treated Hbb^th3^ CD46^+/+^ mice (Figure 9, third panel), whereas 2.7 ± 0.8–fold more blue spots per square centimeter spleen tissue were counted for HDAd-short-LCR– versus HDAd-long-LCR–treated animals (*N* = 5).

In summary, reticulocytes, blood parameters, extracellular hematopoiesis, and hemosiderosis in HDAd-long-LCR–treated animals were not significantly different from control CD46tg mice, indicating a complete phenotypic correction. Furthermore, HDAd-long-LCR proved superior over HDAd-short-LCR in curing thalassemic mice in several phenotypic parameters, most likely due to higher γ-globin levels expressed from the long-LCR.

### Comparison of the 2 vectors after human CD34^+^ transduction and erythroid differentiation.

**Figure 10:**
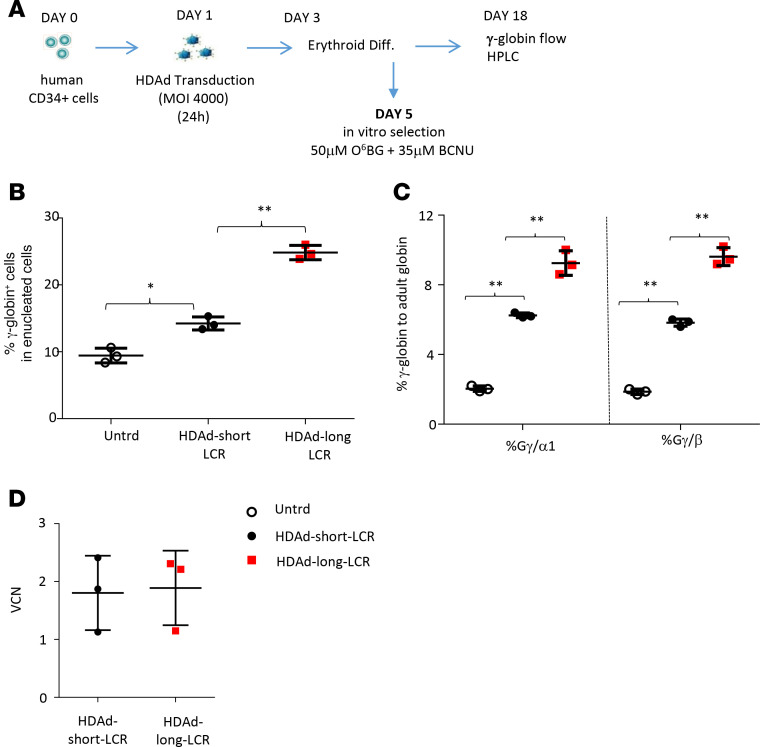
In vitro studies with human CD34^+^ cells. (**A**) Schematic of the experiment: CD34^+^ cells were transduced with HDAd-long-LCR + HD-SB or HDAd-short-LCR + HDAd-SB and subjected to erythroid differentiation (ED). In vitro selection with O^6^BG-BCNU was started at day 5 of ED. At day 18 cells were analyzed by flow cytometry (**B**) and HPLC (**C**). (**D**) VCN at day 18. Statistical analyses were performed using 2-way ANOVA. *P < 0.05; **P < 0.0001.

To consolidate our data in mice, we performed an in vitro study in human cells (Figure 10A). We transduced human CD34^+^ cells obtained from G-CSF–mobilized healthy donors with HDAd-long-LCR + HDAd-SB or HDAd-short-LCR + HDAd-SB at a total MOI of 4000 vp/cells, i.e., a MOI that confers the transduction of the majority of CD34^+^ cells (25). Transduced cells were then subjected to ED and O^6^BG/BCNU selection for cells with integrated transgenes. During expansion of transduced cells over 18 days, most episomal vectors are lost. At the end of ED, we found significantly higher percentages of γ-globin–positive enucleated cells (i.e., reticulocytes that lost the nucleus) by flow cytometry (Figure 10B) and significantly higher γ-globin chain levels by HPLC in the HDAd-long-LCR versus HDAd-short-LCR setting (Figure 10C). The VCN measured at day 18 was approximately 2 for both vectors (Figure 10D).

Our ex vivo and in vivo HSPC transduction studies with mice as well as our in vitro studies with human HSPCs support the relevance of HDAd-long-LCR for gene therapy of hemoglobinopathies.

## Discussion

We are working on the clinical development of an in vivo HSPC gene therapy approach that does not require leukapheresis, myeloablation, and HSPC transplantation (5). These are critical obstacles to a widespread application for ex vivo HSPC gene therapy of hemoglobinopathies, particularly in older patients and patients with comorbidities. We have demonstrated the safety and efficacy of this approach in several murine disease models (8–10) and, recently, in nonhuman primates (11). In both species, we have addressed a major problem associated with intravenous HDAd5/35++ injection, namely acute innate immune responses, by a prophylaxis regimen that blocked proinflammatory cytokines.

Reaching curative γ- or β-globin expression levels in thalassemia major and SCD patients in ex vivo HSPC gene therapy settings is still a challenge. It requires approaches to increase the number of integrated transgene copies by optimizing the HSPC transduction process or by increasing the MOI. Increasing the VCN, however, bears the risk of inducing genotoxicity. Other attempts focus on further optimizing globin expression cassettes (26). With high-payload-capacity HDAd vectors, we have the opportunity to go beyond the vector genome size limitations set for lentivirus and rAAV vectors. In the present study, we demonstrate that curative levels of γ-globin can be achieved in RBCs by in vivo HSPC gene therapy with an integrating HDAd5/35++ vector accommodating β-globin LCR/promoter elements with a total length of 29 kb.

**Figure 11:**
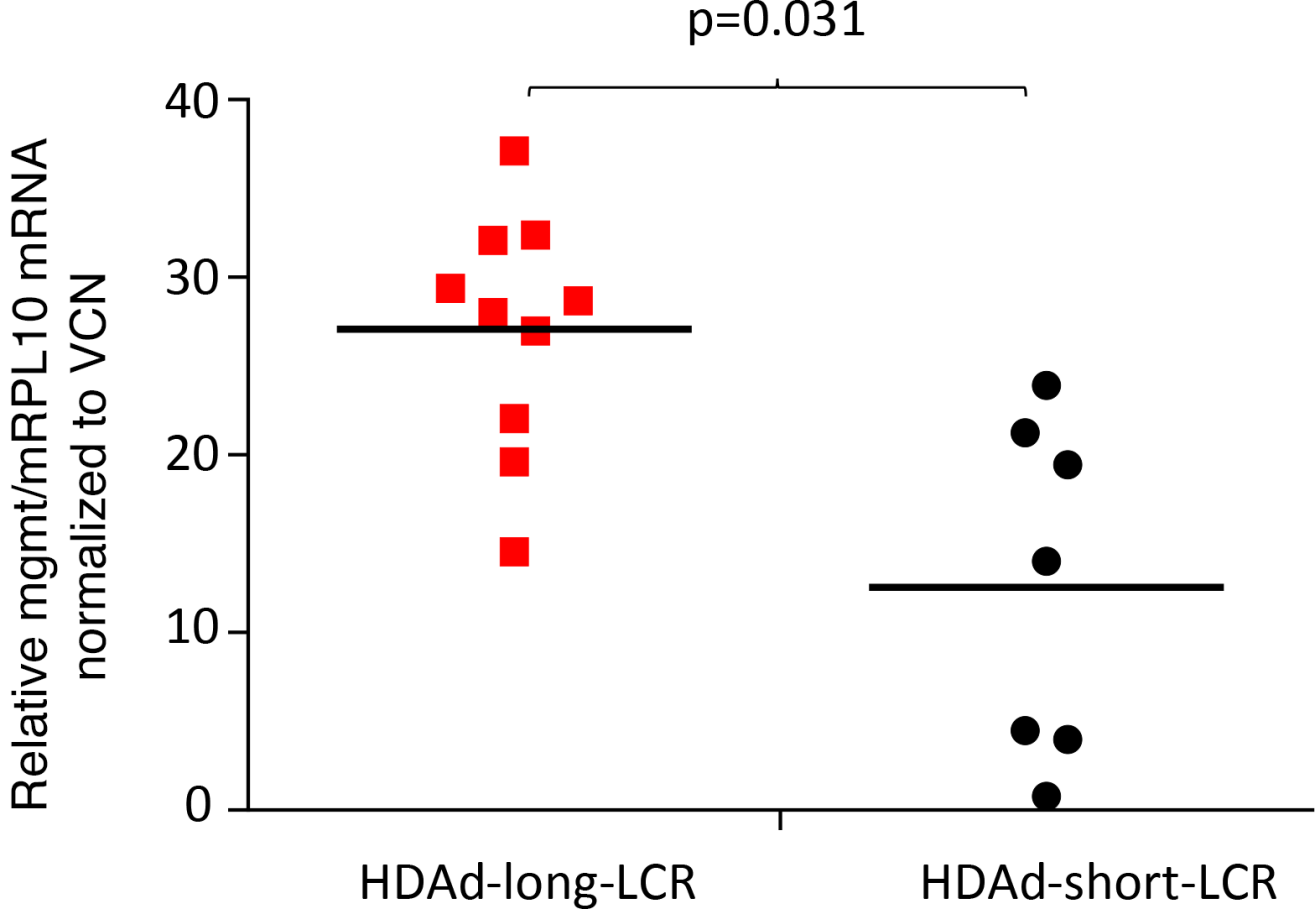
mgmtP140K mRNA expression levels in bone marrow MNCs at week 16 after in vivo transduction. Human mgmt^P140K^ and mouse mRPL10 mRNA levels were measured by qRT-PCR in total bone marrow MNCs. (mRPL10 is a mouse housekeeping gene.) The relative levels were further divided by the VCN (see Supplemental Figure S4). Statistical analyses were performed using 2-way ANOVA.

In thalassemic mice, 100% γ-globin marking in RBCs was achieved earlier and with fewer cycles of O^6^BG/BCNU in vivo selection in mice treated with HDAd-long-LCR compared with HDAd-short-LCR. This is important for the clinical translation of the approach. While the O^6^BG/BCNU in vivo selection system allows for a controlled increase of the percentage of γ-globin–positive RBCs, it also causes transient leukopenia and side effects on the GI tract (8). A potential explanation for the requirement of less intense in vivo selection with HDAd-long-LCR could be that the long-LCR prevents silencing of the EF1α promoter driving the expression of the mgmt^P140K^ gene that provides resistance to O^6^BG/BCNU. This hypothesis is supported by the observation that mgmt mRNA levels (normalized to VCN) in bone marrow MNCs were significantly higher for HDAd-long-LCR (Figure 11).

**Figure 12:**
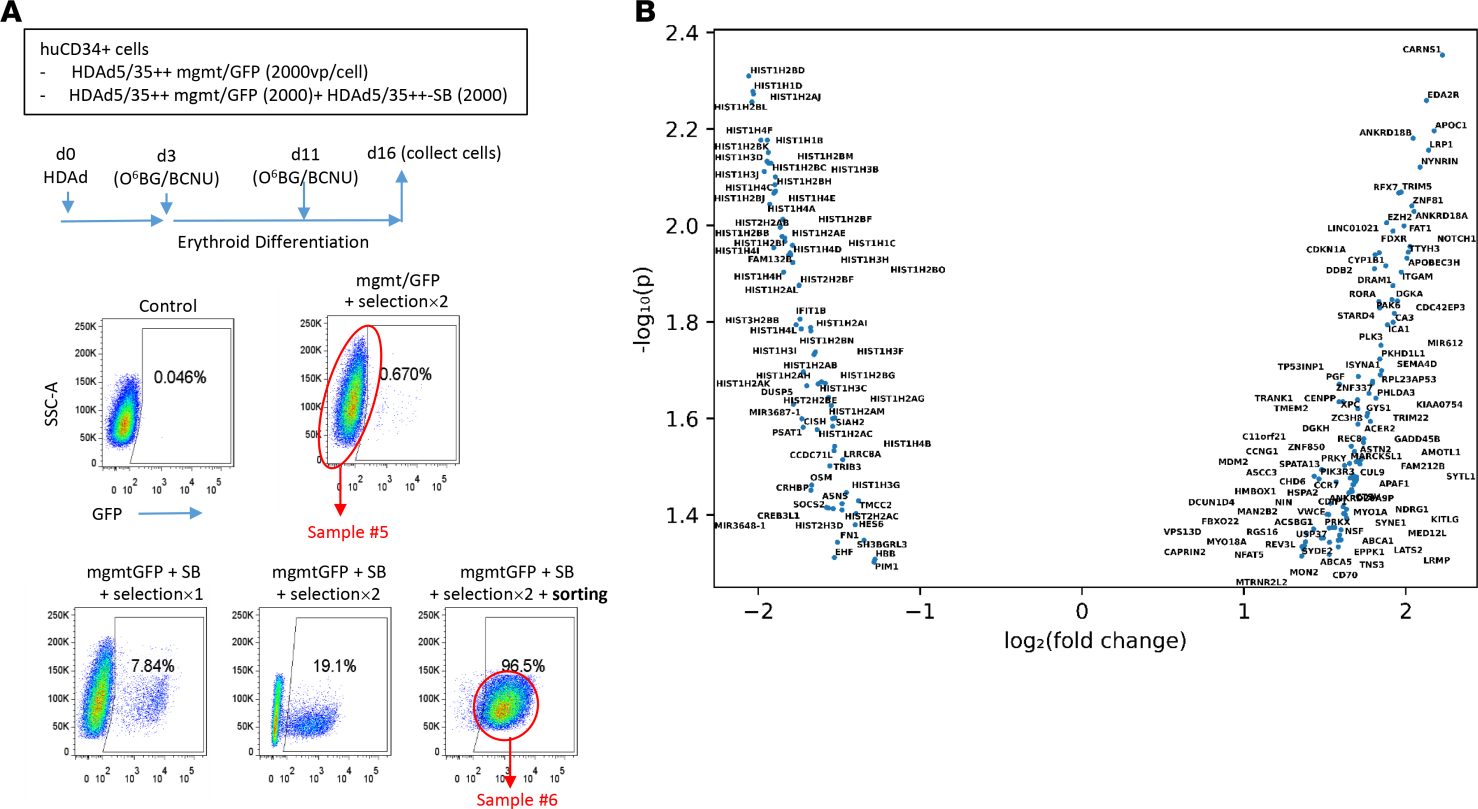
Effect of SB100x-mediated integration on the transcriptome of CD34^+^ cells. (**A**) Schematic of experiment. CD34^+^ cells were infected with a HDAd5/35++ vector containing a GFP/mgmt cassette under control of the EF1α promoter alone or in combination with HDAd-SB. Transduced cells were expanded in ED medium for 16 days. Two rounds of O^6^BG/BCNU selection (50 μM O^6^BG + 35 μM BCNU) were enriched for GFP-positive cells with integrated transposons. At day 16, GFP-positive cells were FACS sorted (sample 6). For comparison (sample 5), CD34^+^ cells that were transduced with the mgmt/GFP vector alone and subjected to selection were used. Because the control cells did not express SB100x, they lost the episomal mgmt/GFP vector and were therefore GFP negative. Total RNA from both samples was subjected to RNA-Seq performed by Omega Bioservices. (**B**) Genes with altered mRNA expression (log_2_ fold change) ranked based on their *P* value.

While we focused in this study on therapeutic aspects of our in vivo approach using HDAd-long-LCR, a number of mechanistic questions remain to be addressed in the future. One of these open questions is whether the long-LCR prevents the transactivation of distant and neighboring genes. Furthermore, it is not completely clear whether the higher γ-globin expression levels from HDAd-long-LCR, which are also reflected at the mRNA level, are due to more active transcription initiation, less silencing of integrated vector copies, or both. The observation that in HDAd-long-LCR–treated Hbb^th3^ CD46 mice, the percentage of γ-globin to mouse adult globin chains increased over time, a phenomenon that was also seen with in the CD46tg model in secondary recipients, could indicate that silencing, specifically in long-term repopulating cells, occurred over time and that the long-LCR protected against it. As outlined above, higher mgmt^P140K^ mRNA levels per integrated vector copy (Figure 11) also support the hypothesis that the long-LCR protects against silencing. To address these questions, in future studies, we will focus on transduced CD34^+^ cell clones and perform genome-wide analysis using LAM-PCR/next-generation sequencing (integration sites), chromosome conformation capture techniques, and RNA-Seq. A prerequisite for these studies would be that the SB100x transposase–mediated transgene integration and in vivo selection processes do not trigger undesired genomic alterations/rearrangements. In an attempt to assess this, we performed RNA-Seq on human CD34^+^ cells that stably expressed mgmt/GFP transgenes after SB100x-mediated integration and O^6^BG/BCNU selection in vitro (Figure 12A). We found modestly altered expression of only 176 genes, preferentially histone genes (Figure 12B and Supplemental Data). This indicates that SB100x does not exert critical genotoxicity, which is also supported by the absence of clonal dominance in integration site analysis and the absence of hematological side effects in long-term studies.

The copy number of integrated transgenes analyzed in bone marrow MNCs 16 to 23 weeks after in vivo HSPC transduction/selection using the HDAd5/35++-based SB100x system was approximately 2 copies per cell for transposons ranging from 13.8 (9) to 32.4 kb. In order to form a catalytically primed transposon/transposase complex, the 2 ends of the transposon must be held together in close physical proximity by transposase molecules (27). This limitation has been addressed by incorporating frt sides into the HDAd vector, which are recognized by the coexpressed Flpe recombinase, leading to a circularization of the transposon (16). Our data suggest that this process may make integration largely independent of the size of the transposon carried by HDAd5/35++ vectors.

Our study demonstrates that using extended TAD/LCR core elements increases the expression level of a therapeutic transgene. While the β-globin LCR has been studied for decades, TAD core elements for other genes/clusters are less characterized. The median size of TAD is 880 kb. With further advancement of high-throughput chromosome conformation capture (3C) assay and its subsequent 4C, 5C, and Hi-C protocols as well as fiber-Seq assays, the interrogation of the regulatory genome will progress at a rapid speed and, for gene therapy purposes, could deliver TADs that contain only critical core elements (2).

In summary, by employing large regulatory elements in the context of HDAd5/35++ vectors for in vivo transduction of HSPCs, we were able to reach γ-globin levels in erythroid cells that completely cured murine thalassemia intermedia. Our efficacy and safety data provide a basis for a potential clinical translation of the approach.

## Methods

### HDAd vectors

The generation of HDAd-SB and HDAd-short-LCR vector has been described previously (5, 13). For the generation of the HDAd-long-LCR vector, corresponding shuttle plasmids were based on the cosmid vector pWE15 (Stratagene). pWE.Ad5-SB-mgmt contains the Ad5 5′ITR (nucleotides 1 through 436) and 3′ITR (nucleotides 35741 through 35938), the human EF1α promoter-mgmt(p140k)-SV-40pA-cHS4 cassette derived from pBS-μLCR-γ-globin-mgmt (8), and SB100x-specific IR/DR sites and FRT sites. The GFP-BGHpA fragment in the pAd.LCR-β-GFP (contains a 21.5 kb human β-globin LCR (28) was replaced by the human γ-globin gene and its 3′UTR region (chr11:5,247,139 → 5,249,804) (a gift from Qiliang Li, University of Washington, Seattle, Washington, USA) (pAd-long-LCR-β-γ-globin). The plasmid pAd-long-LCR-β-γ-globin contains a 21.5 kb human β-globin LCR and 3.0 kb human β-globin 3′HS1. The 28.9 kb fragment containing LCR-β-γ-globin-3′HS1 was inserted downstream of the cassette of EF1α-mgmt-SV-40pA-cHS4 into pWE.Ad5-SB-mgmt (pWE.Ad5-SB-long-LCR-γ-globin/mgmt). The complete long-LCR-γ-globin/mgmt cassette was flanked by SB100x-specific IR/DR sites and FRT sites. The resulting plasmids were packaged into phages using Gigapack III Plus Packaging Extract (Stratagene) and propagated. To generate the HDAd-long-LCR-γ-globin/mgmt virus, the viral genomes were released by I-CeuI digestion from the plasmid for rescue in 116 cells. There are 2 known variants of the HBG1 gene in the human population with a single amino acid variation (76-isoleucine or 76-threonine). We used in our studies the 76-Ile HBG1 variant, which has a range in frequency from 13% in Europeans to 73% in East Asians.

The Ad5/35++-Acr helper virus is a derivative of AdNG163-5/35++, an Ad5/35++ helper vector containing chimeric fibers composed of the Ad5 fiber tail, the Ad35 fiber shaft, and the affinity-enhanced Ad35++ fiber knob (5). A human codon-optimized AcrIIA4-T2A-AcrIIA2 sequence that was recently shown to inhibit SpCas9 activity was synthesized (25) and subcloned into a shuttle plasmid pBS-CMV-pA (pBS-CMV-Acr-pA). Subsequently, the 2.0 kb CMV-Acr-pA cassette was amplified from pBS-CMV-Acr-pA and inserted into the SwaI sites of pNG163-2-5/35++ (5) by In-Fusion HD cloning kit (Takara). The viral genome was then released by PacI digestion, and the Ad5/35++-Acr helper virus was rescued and propagated in HEK293 (ATCC CRL-1573) cells. The generation of HDAd-SB has been described previously (5). Helper virus contamination levels were below 0.05%. All preparations were free of bacterial endotoxin.

### CD34^+^ cell culture

CD34^+^ cells from G-CSF–mobilized adult donors were recovered from frozen stocks and incubated overnight in Iscove’s modified Dulbecco’s medium (IMDM) supplemented with 10% heat-inactivated FCS, 1% BSA 0.1 mmol/L 2-mercaptoethanol, 4 mmol/L glutamine and penicillin/streptomycin (pen/strep), Flt3 ligand (Flt3L, 25 ng/mL), IL-3 (10 ng/mL), thrombopoietin (TPO) (2 ng/mL), and stem cell factor (SCF) (25 ng/mL). Flow cytometry demonstrated that more than 98% of cells were CD34^+^. Cytokines and growth factors were from PeproTech. CD34^+^ cells were transduced with virus in low-attachment 12-well plates.

### Erythroid in vitro differentiation

Differentiation of human HSPCs (from the Hematopoietic Cell Procurement and Processing Services Lab at the Fred Hutchinson Cancer Research Center, Seattle, Washington, USA) into erythroid cells was done based on the protocol developed by Douay et al. (29). In brief, in step 1, cells at a density of 10^4^ cells/mL were incubated for 7 days in IMDM supplemented with 5% human plasma, 2 IU/mL heparin, 10 μg/mL insulin, 330 μg/mL transferrin, 1 μM hydrocortisone, 100 ng/mL SCF, 5 ng/mL IL-3, 3 U/mL erythropoietin (Epo), glutamine, and pen/strep. In step 2, cells at a density of 1 × 10^5^ cells/mL were incubated for 3 days in IMDM supplemented with 5% human plasma, 2 IU/mL heparin, 10 μg/mL insulin, 330 μg/mL transferrin, 100 ng/mL SCF, 3 U/mL Epo, glutamine, and pen/strep. In step 3, cells at a density of 1 × 10^6^ cells/mL cells were incubated for 12 days in IMDM supplemented with 5% human plasma, 2 IU/mL heparin, 10 μg/mL insulin, 330 μg/mL transferrin, 3 U/mL Epo, glutamine, and pen/strep.

### In vitro selection of transduced CD34^+^ cells

Transduced CD34^+^ cells were selected with O^6^BG/BCNU on day 5 in step 1 of the in vitro differentiation protocol. Briefly, CD34^+^ cells were incubated with 50 μM O^6^BG for 1 hour and then incubated with 35 μM BCNU for another 2 hours; cells were then washed twice and resuspended in fresh step 1 medium.

### Lin^–^ cell culture

Lin^–^ cells were isolated form total mouse bone marrow cells by MACS using the Lineage Cell Depletion kit from Miltenyi Biotec. Lin^–^ cells were cultured in IMDM supplemented with 10% FCS, 10% BSA, pen/strep, glutamine, 10 ng/mL human TPO, 20 ng/mL mouse SCF, and 20 ng/mL human Flt3L.

### Globin HPLC

Individual globin chain levels were quantified on a Shimadzu Prominence instrument with an SPD-10AV diode array detector and an LC-10AT binary pump (Shimadzu). A 40%–60% gradient mixture of 0.1% trifluoroacetic acid in water/acetonitrile was applied at a rate of 1 mL/min using a Vydac C4 reversed-phase column (Hichrom).

### Flow cytometry

Cells were resuspended at 1 × 10^6^ cells/100 μL in PBS supplemented with 1% FCS and incubated with FcR blocking reagent (Miltenyi Biotec) for 10 minutes on ice. Next the staining antibody solution was added in 100 μL per 10^6^ cells and incubated on ice for 30 minutes in the dark. After incubation, cells were washed once in FACS buffer (PBS, 1% FBS). The staining step was repeated with a secondary staining solution. After the wash, cells were resuspended in FACS buffer and analyzed using an LSRII flow cytometer (BD Biosciences). Debris was excluded using a forward scatter area and sideward scatter area gate. Single cells were then gated using a forward scatter height and forward scatter width gate. Flow cytometry data were then analyzed using FlowJo (version 10.0.8, FlowJo, LLC). For flow analysis of Lin^–^Sca1^+^c-Kit^+^ cells, cells were stained with biotin-conjugated lineage detection cocktail (Miltenyi Biotec) (130-092-613) and antibodies against c-Kit (12-1171-83) and Sca-1 (25-5981-82) as well as APC-conjugated streptavidin. Other antibodies from eBioscience (Thermo Fisher Scientific) included anti–mouse LY-6A/E (Sca-1)-PE-cyanine7 (clone D7), anti–mouse CD117 (c-Kit)-PE (clone 2B8), anti–mouse CD3-APC (clone 17A2) (17-0032-82), anti–mouse CD19-PE-cyanine7 (clone eBio1D3) (25-0193-82), and anti–mouse Ly-66 (Gr-1)-PE (clone RB6-8C5) (12-5931-82). Anti–mouse Ter-119-APC (clone Ter-119, catalog 116211) was from BioLegend.

### Intracellular flow cytometry detecting human γ-globin expression

The FIX & PERM cell permeabilization kit (Thermo Fisher Scientific) was used and the manufacturer’s protocol was followed. Briefly, approximately 1 × 10^6^ cells were resuspended in 100 μL FACS buffer (PBS supplemented with 1% FCS); 100 μL of reagent A (fixation medium) was added and incubated for 2–3 minutes at room temperature; and 1 mL precooled absolute methanol was then added, mixed, and incubated on ice in the dark for 10 minutes. The samples were then washed with FACS buffer and resuspended in 100 μL reagent B (permeabilization medium) and 0.3 μg hemoglobin-γ antibody (Santa Cruz Biotechnology, sc-21756 PE), incubated for 30 minutes at room temperature. After the wash, cells were resuspended in FACS buffer and analyzed. Flow cytometry gating strategies are shown in Supplemental Figures 6 and 7.

### Real-time reverse transcription PCR

Total RNA was extracted from 50~100 μL blood by using TRIzol reagent (Thermo Fisher Scientific) following the manufacturer’s phenol/chloroform extraction method. Quantitect reverse transcription kit (QIAGEN) and *Power* SYBR Green PCR Master Mix (Applied Biosystems, Thermo Fisher Scientific) were used. Real-time qPCR was performed on a StepOnePlus real-time PCR system (Applied Biosystems, Thermo Fisher Scientific). The following primer pairs were used: mouse RPL10 (housekeeping) forward, 5′-TGAAGACATGGTTGCTGAGAAG-3′, and reverse, 5′-GAACGATTTGGTAGGGTATAGGAG-3′; human γ-globin forward, 5′-GTGGAAGATGCTGGAGGAGAAA-3′, and reverse, 5′-TGCCATGTGCCTTGACTTTG-3′; mouse β-major globin forward, 5′-ATGCCAAAGTGAAGGCCCAT-3′, and reverse, 5′-CCCAGCACAATCACGATCAT-3′, mouse α-globin forward, 5′-ctggggaagacaaaagcaac-3′, and reverse, 5′-gccgtggcttacatcaaagt-3.

### Measurement of VCN

Total DNA from bone marrow cells was extracted using the *Quick*-DNA Miniprep kit (Zymo Research). Viral DNA extracted from HDAd-short LCR-γ-globin/mgmt virus was serially diluted and used for a standard curve. qPCR was conducted in triplicate using the *Power* SYBR Green PCR Master Mix on a StepOnePlus real-time PCR system. For a 10 μL reaction 9.6 ng DNA (9600 pg/6 pg/cell = ~1600 cells) was used. The following primer pairs were used: human γ-globin forward, 5′-gtgcttgaaggggaacaactac-3′, and reverse, 5′-cctggcctccagataactacac-3′.

### Integration site analysis

For a description of the procedure, please see Supplemental Figure 3. The randomized data for Figure 4D were created using a Poisson regression insertion model (PRIM) to calculate the expected insertion rate for nonoverlapping 20 kb windows along the length of each chromosome in the mouse reference genome (mm9). The PRIM algorithm generated a statistical model based on the number of TA dinucleotides within each window, the chromosome in which the window resides, and the total number of unique insertions. For each window, the expected number of insertions was calculated and compared with the observed number of insertions to produce a *P* value. Bonferroni’s correction was then applied to identify windows that showed enrichment for detection of inserted transposons. Random sequences from the reference genome containing TA were then generated, mapped using Bowtie2, and plotted against our real integration data. Calculations and plots were made using ggplot2 in R. Figures were drawn using HOMER and ChIPseeker.

### Integration site analysis (iPCR)

Junctions in total bone marrow cells were analyzed by iPCR as described elsewhere with modifications (28). Briefly, genomic DNA from bone marrow cells was isolated by *Quick*-DNA Miniprep kit following the manufacturer’s instructions. Next 5~10 μg of DNA was digested with SacI and religated under conditions that promote intramolecular reaction. The ligation mixture was purified with phenol/chloroform extraction and ethanol precipitation and then used for nested PCR (30 cycles each) using KOD Hot Start DNA polymerase (MilliporeSigma). The following primers were used: EF1α p1 forward, 5′-ccccctcgaggtcgacatggctagagacttatcgaaagca-3′, and reverse, 5′-attcgatatcaagctccaagatctgcacactggtattt -3′; EF1α p2 forward, 5′-ccccctcgaggtcgacgtacacgacatcactttcccagt -3′, and reverse, 5′-attcgatatcaagctcacactggtatttcggtttttg-3′; 3′HS1 p1 forward, 5′-ccccctcgaggtcgacctacactctcagtcagcctatgga-3′, and reverse, 5′-attcgatatcaagcttaatcccaaaaggctgatagtctc-3′. 3′HS1 p2 forward, 5′-ccccctcgaggtcgacacatctctcactttctcatcacca-3′, and reverse, 5′-attcgatatcaagctaagtaactgggattacaggagcac-3′. The underlined bases were used for downstream cloning. PCR amplicons were gel purified, cloned, sequenced, and aligned to identify the integration sites.

### RNA-Seq analysis

RNA-Seq analysis was performed by Omega Bioservices (Norcross, Georgia, USA). Data were analyzed by Rosalind (https://rosalind.onramp.bio/), with a HyperScale architecture developed by OnRamp BioInformatics, Inc. Reads were trimmed using cutadapt. Quality scores were assessed using FastQC. Individual sample reads were quantified using HTseq4 and normalized via relative log expression using DESeq2 R library. DESeq2 was also used to calculate fold changes and *P* values and perform optional covariate correction. Enrichment was calculated relative to a set of background genes relevant for the experiment. The volcano plot was generated with custom a Python script that plots log-scale fold change versus *P* values, and only genes meeting significance *P* < 0.01 are displayed. RNA-Seq data were deposited into the National Center for Biotechnology Information’s Gene Expression Omnibus database (GSE155843).

### Animals

Ex vivo and in vivo HSPC transduction studies were performed with a C57BL/6-based transgenic mouse model (hCD46tg) that contained the complete hCD46 locus (20). These mice express hCD46 in a pattern and at a level similar to humans. Hbb^th3^ (B6.D2-*Hbb^d3th^*/BrkJ) and C57BL/6 mice were from The Jackson Laboratory.

#### Breeding and screening of Hbb^th3^ CD46^+/+^ mice.

After 3 rounds of backcrossing, Hbb^th3^ CD46^+/+^ mice’s homozygosity for CD46 was confirmed by PCR on genomic DNA (using CD46F-5′-AAAGGGCAAATTACCTTAAGGGGTG-3′ and CD46R-5′-AGCACTTCGACCTAAAAATAGAGAT-3′ primers) as well as by flow cytometry that allowed measuring CD46 MFI. The thalassemic phenotype of Hbb^th3^ CD46^+/+^ mice was assessed by peripheral blood smears, after Giemsa/May-Grünwald staining, as described below.

#### Bone marrow Lin^–^ cell transplantation.

Recipients were female C57BL/6 mice, 6–8 weeks old. On the day of transplantation, recipient mice were irradiated with 10 Gy. Four hours after irradiation 1 × 10^6^ Lin^–^ cells were injected intravenously through the tail vein. This protocol was used for transplantation of ex vivo transduction Lin^–^ cells and for transplantation into secondary recipients.

#### HSPC mobilization and in vivo transduction.

This procedure was described previously (5). Briefly, HSPCs were mobilized in mice by s.c. injections of human recombinant G-CSF (5 μg/mouse/d, 4 days) (Amgen) followed by an s.c. injection of AMD3100 (5 mg/kg) (MilliporeSigma) on day 5. In addition, animals received dexamethasone (10 mg/kg) i.p. 16 hours and 2 hours before virus injection. Thirty and 60 minutes after AMD3100, animals were intravenously injected with HDAd vectors through the retro-orbital plexus with a dose of 4 × 10^10^ vp for each virus per injection. Four weeks later, in vivo selection of O^6^BG/BCNU was initiated as described in the figure legends.

### Secondary bone marrow transplantation

Recipients were female C57BL/6 mice, 6–8 weeks old, from The Jackson Laboratory. On the day of transplantation, recipient mice were irradiated with 10 Gy. Bone marrow cells from in vivo–transduced CD46tg mice were isolated aseptically, and lineage-depleted cells were isolated using MACS. Four hours after irradiation cells were injected intravenously at 1 × 10^6^ cells per mouse. All secondary recipients received immunosuppression starting at week 4.

### Tissue analysis

Spleen and liver tissue sections of 2.5 μm thickness were fixed in 4% formaldehyde for at least 24 hours, dehydrated, and embedded in paraffin. Staining with H&E was used for histological evaluation of extramedullary hemopoiesis. Hemosiderin was detected in tissue sections by Perls’ Prussian blue staining. Briefly, the tissue sections were treated with a mixture of equal volumes (2%) of potassium ferrocyanide and hydrochloric acid in distilled water and then counterstained with neutral red. To quantitate extracellular hemopoiesis and hemosiderosis, 10 random areas in 5 different tissue sections from at least 3 animals were evaluated by investigators who were blinded to the mouse groups. The spleen size was assessed as the ratio of spleen weight (mg)/body weight (g).

### Blood analysis and bone marrow cytospins

Blood samples were collected into EDTA-coated tubes, and analysis was performed on a HemaVet 950FS (Drew Scientific). Peripheral blood smears and bone marrow cell cytospins were stained with Giemsa/May-Grünwald (Merck) for 5 and 15 minutes, respectively. Reticulocytes were stained with Brilliant cresyl blue. The investigators who counted the reticulocytes on blood smears were blinded to the sample group allocation. Only animal numbers appeared on the slides (5 slides per animal, 5 random 1 cm^2^ sections).

### Statistics

Data are presented as mean ± SEM. For comparisons of multiple groups, 1-way and 2-way ANOVA with Bonferroni’s posttesting for multiple comparisons were employed. Differences between groups for 1 grouping variable were determined by the unpaired, 2-tailed Student’s *t* test. For nonparametric analyses, the Kruskal-Wallis test was used. Statistical analysis was performed using GraphPad Prism version 6.01 (GraphPad Software Inc.). *P ≤ 0.05; **P ≤ 0.0001. A *P* value less than 0.05 was considered significant.

### Study approval

All experiments involving animals were conducted in accordance with the institutional guidelines set forth by the University of Washington. The University of Washington is an Association for the Assessment and Accreditation of Laboratory Animal Care International–accredited research institution, and all live animal work conducted at this university is in accordance with the Office of Laboratory Animal Welfare Public Health Assurance policy, USDA Animal Welfare Act and regulations, the *Guide for the Care and Use of Laboratory Animals* (National Academies Press, 2011), and the University of Washington’s Institutional Animal Care and Use Committee (IACUC) policies. The studies were approved by the University of Washington IACUC (protocol 3108-01).

## Author contributions

AL provided the conceptual framework for the study. HW and AG designed the experiments. HW, AG, ZL, CL, and SG performed the experiments. AP and ZI performed the integration analyses. AB helped with the transcriptome analysis. CL, EY, AA, and TP provided comments. AL wrote the manuscript.

## Supplementary Material

Supplemental data

## Version 1 08/20/2020

Electronic publication
