# Supplementary material for: Curative in vivo hematopoietic stem cell gene therapy of murine thalassemia using large regulatory elements
Source: JCI Insight. 2020 Aug 20;5(16):e139538. doi: 10.1172/jci.insight.139538 (PMC7455141; doi:10.1172/jci.insight.139538)
Supplement: Supplemental data [file jciinsight-5-139538-s059.pdf]

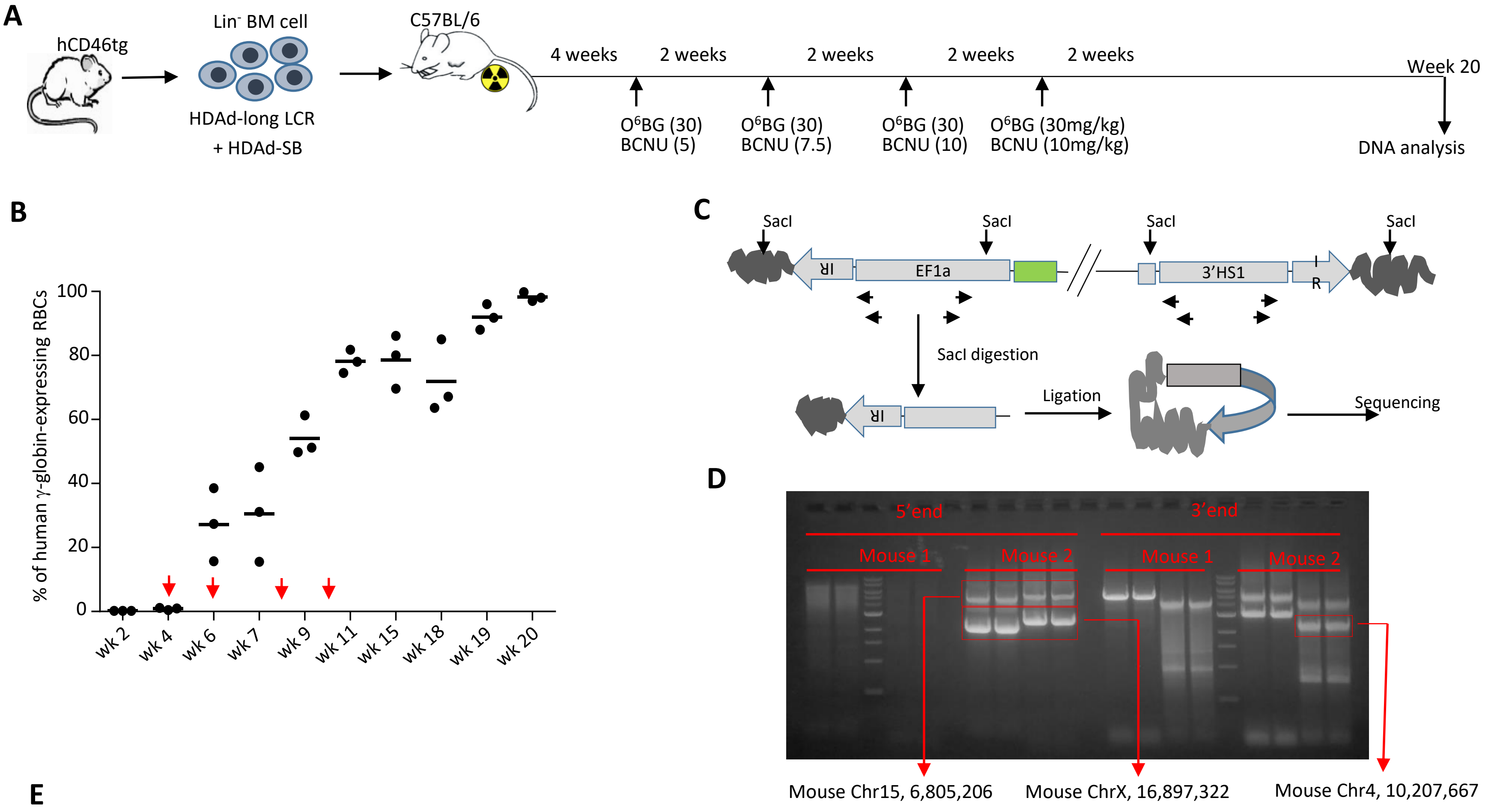

E

CAGTCAACTT AGTGTATGTA AACTTCTGAC CCACTGGAAT TGTGATACAG TGAATTATAA GTGAAATAAT CTGTCTGTAA
ACAATTGTTG GAAAAATGAC TTGTGTCATG CACAAAGTAG ATGTCCTAAC TGACTTGCCA AAACTATTGT TTGTTAACAA
GAAATTTGTG GAGTAGTTGA AAAACGAGTT TTAATGACTC CAACTTAAGT GTATGTAAAC TTCCGACTTC AACTG**TA**AGA
ATGGCCCATT CATCTATAGT AGCACACAAT ATTTGCATTT GTGCGACAGT ATAAGGGACA ATTATGCTAT CAGGCATTTT
TCCAAAGTGA GTAATCGAAG TTTTTATACC TTTGTGTGCC ATGTTTGCTA

5'end vector sequence
Sleeping beauty IR/DR sequence
Integration junction (chr15, 6805206)

CAGTCAACT TAGTGTATGT AAACTTCTGA CCCACTGGAA TTGTGATACA GTGAATTATA AGTGAAATAA TCTGTCTGTA
AACAATTGTT GGAAAAATGA CTTGTGTCAT GCACAAAGTA GATGTCCTAA CTGACTTGCC AAAACTATTG TTTGTTAACA
AGAAATTTGT GGAGTAGTTG AAAAACGAGT TTTAATGACT CCAACTTAAG TGTATGTAAA CTTCCGACTT CAACTG**TA**CA
AGTAGACCAA ATATCCATAT ACATAAAAGA AAAAAATAGA AAAAATTTCT AGTGACAGAA AAATGACAAA GAACATACTG
CTTTATTACT ACTATTAAGA TGTTTGCTTC CATTACACTC ATATGAGTCA

5'end vector sequence
Sleeping beauty IR/DR sequence
Integration junction (chrX, 16897322)

CAACTTGAGTG TATGTTAACT TCTGACCCAC TGGGAATGTG ATGAAAGAAA TAAAAGCTGA AATGAATCAT TCTCTCTACT
ATTATTCTGA TATTTCACAT TCTTAAAATA AAGTGGTGAT CCTAACTGAC CTTAAGACAG GGAATCTTTA CTCGGATTAA
ATGTCAGGAA TTGTGAAAAA GTGAGTTTAA ATGTATTTGG CTAAGGTGTA TGTAAACTTC CGACTTCAAC TG**TA**TATCCT
CCCCGTTGCA CCCTCTTGAT GATGCTGAGA TGAACACAGA TGCTCACTCC TTGAGGGCTC TAAGCTTATG CTGACACAGA
CACAGGTGCT CACTTCTATG AATGGCCTAA GATTTGAGGA CATCATGAGG

3'end vector sequence
Sleeping beauty IR/DR sequence
Integration junction (chr4, 10207667)

Figure S1. SB100x-mediated integration of the 32.4kb transposon after e*x vivo* HSPC transduction study with HDAd-long-LCR. A) Experimental regimen: Bone marrow Lin$^-$ cells from CD46-transgenic mice were transduced with HDAd-long-LCR and HDAd-SB at a total MOI of 500vp/cell. After one day in culture, 1x10^6 transduced cells/mouse were transplanted into lethally irradiated C57Bl/6 mice. At week 4, O^{6}BG/BCNU treatment was started and repeated four times every two weeks. With each cycle, the BCNU concentration was increased from 5mg/kg, to 7.5mg/kg, to 10mg/kg (twice). At week 20, mice were sacrificed. **B)** Percentage of human γ-globin-positive peripheral red blood cells (RBC) measured by flow cytometry. Each symbol is an individual animal. **C)** Schematic of iPCR analysis: Five micrograms of genomic DNAs were digested with SacI, religated, and subjected to nested, inverse PCR with the indicated primers (see Materials and Methods). **D)** Agarose gel electrophoresis of cloned plasmids containing integration junctions. Indicated bands were excised and sequenced. The chromosomal localization of integration sites are shown below the gel. **E)** Examples for junction sequences. The vector body and IR/DR sequences are in blue and red, respectively. The chromosomal sequence is in green. The TA dinucleotides used by SB100x at the junction of the IR and chromosomal DNA are underlined.

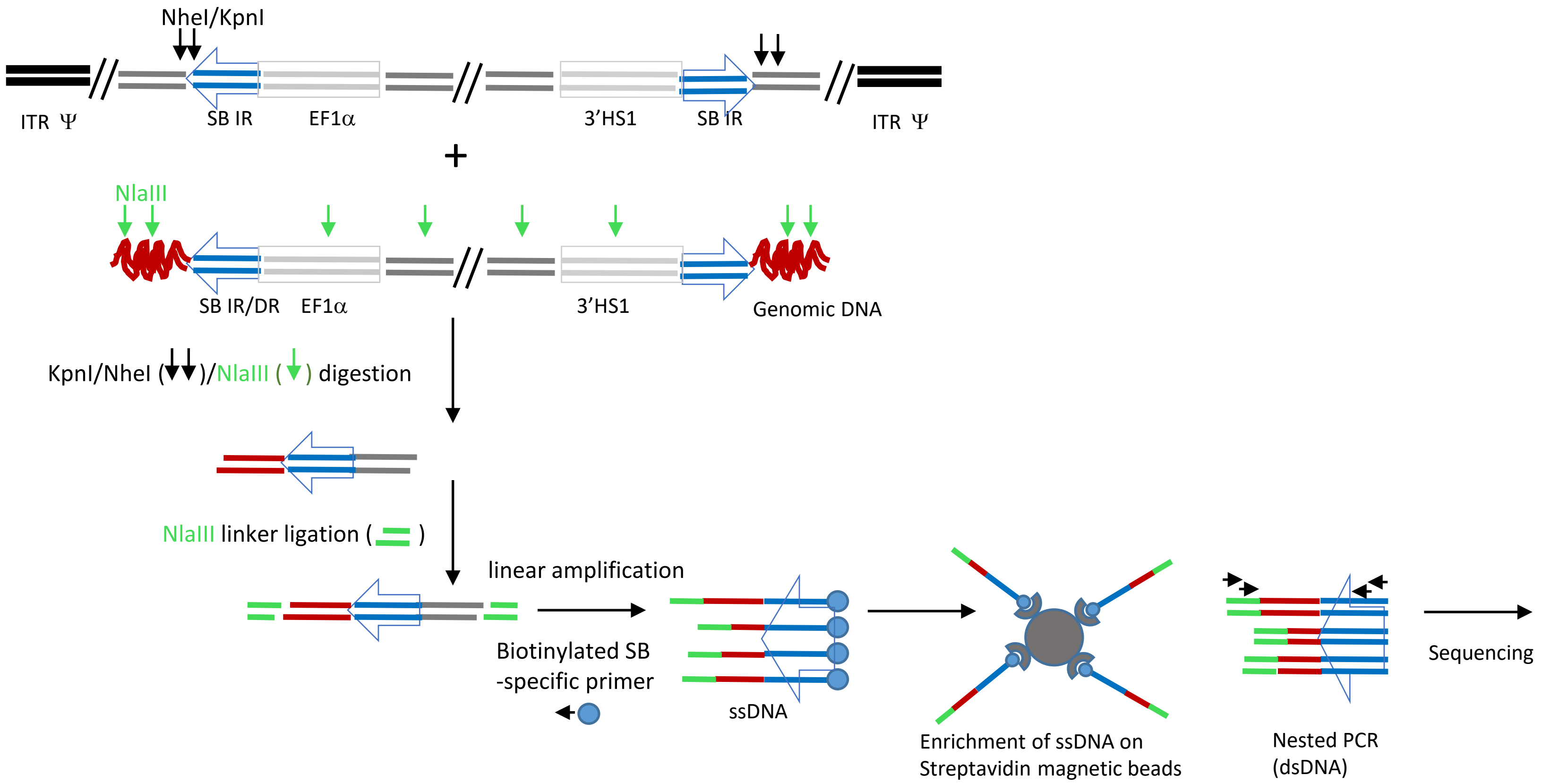

Figure S2: Schematic of insertion site analysis. The localization of NheI and KpnI sites in the HDAd-long-LCR vector in relation to the *Sleeping Beauty* inverted repeats (IRs) is indicated. These enzymes cut close, but outside of the SB IR/DR and are used to decrease the background of unintegrated vectors. Genomic DNA from bone marrow Lin^- cells was digested with NheI and KpnI, and after heat inactivation, further digested with NlaIII. NlaIII is a 4-cutter and will create small DNA fragments. Digested DNA was then ligated with double stranded oligos with known sequence and compatible ends to the digested NlaIII fragments. Following heat-inactivation and clean-up, the linker-ligated products were used for linear amplification, which creates a single-stranded (ss) DNA population primed from the SB left arm. The primer is biotinylated, so the ssDNAs can be collected with streptavidin beads. After extensive washing, ssDNA was eluted from the beads and subjected to further amplification by two rounds of nested PCR. PCR amplicons were gel purified, cloned, sequenced and mapped to the mouse genome sequences to mark the integration sites.

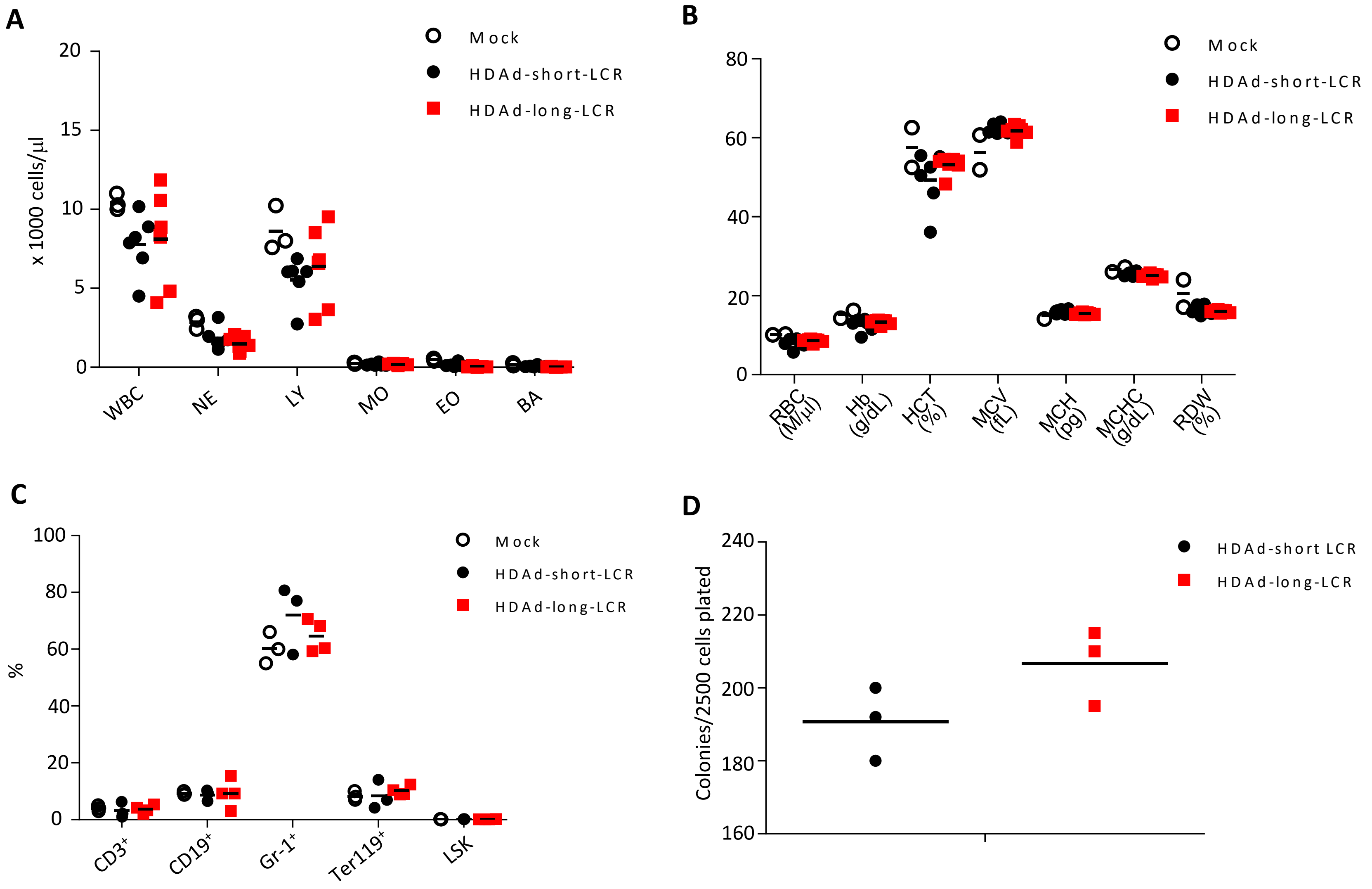

Figures S3. Hematological parameters in secondary recipients at week 16 after transplantation. A) White blood cells. **B)** Erythropoietic parameters. RBC: red blood cells, Hb: hemoglobin, MCV: mean corpuscular volume, MCH: mean corpuscular hemoglobin, MCHC: mean corpuscular hemoglobin concentration, RDW: red cell distribution width. **C)** Cellular bone marrow composition. **D)** Colony-forming potential of bone marrow Lin^- cells. The differences between the groups were not significant in A-D. Statistical analyses were performed using two-way ANOVA.

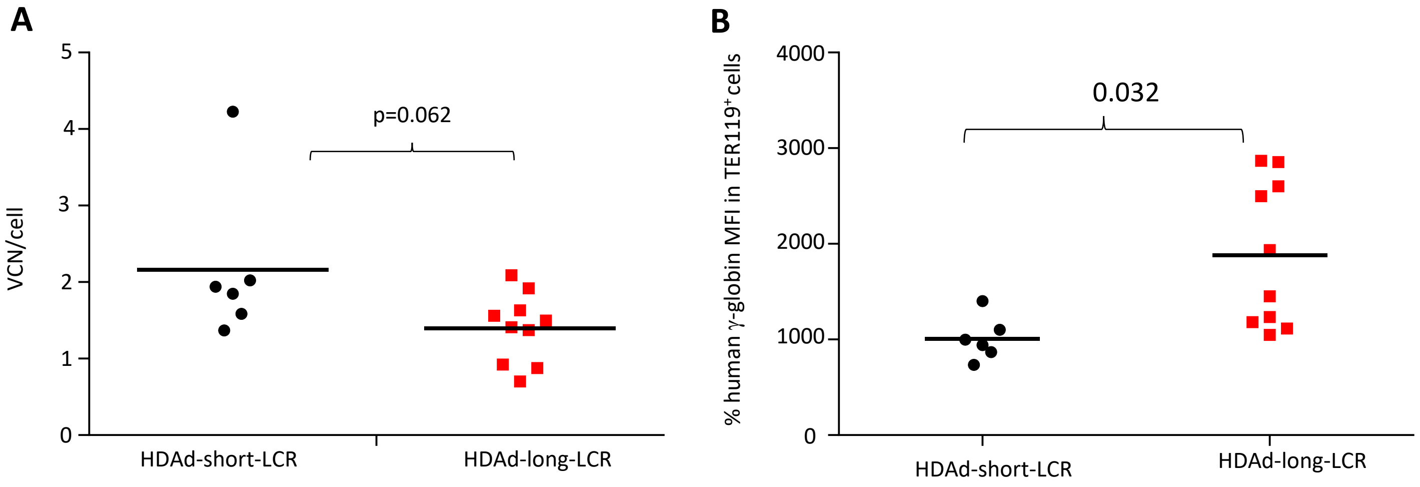

Figure S4. Analysis of bone marrow at sacrifice. Bone marrow was harvested at week 16 after *in vivo* HSPC transduction of $Hbb^{th3}/CD46^{+/+}$ mice. **A)** Vector copy number per cell in bone marrow MNCs. The difference between the two groups is not significant. **B)** Mean Fluorescence Intensity (MFI) of γ-globin in erythroid ($Ter119^{+}$) cells. Statistical analyses were performed using two-way ANOVA.

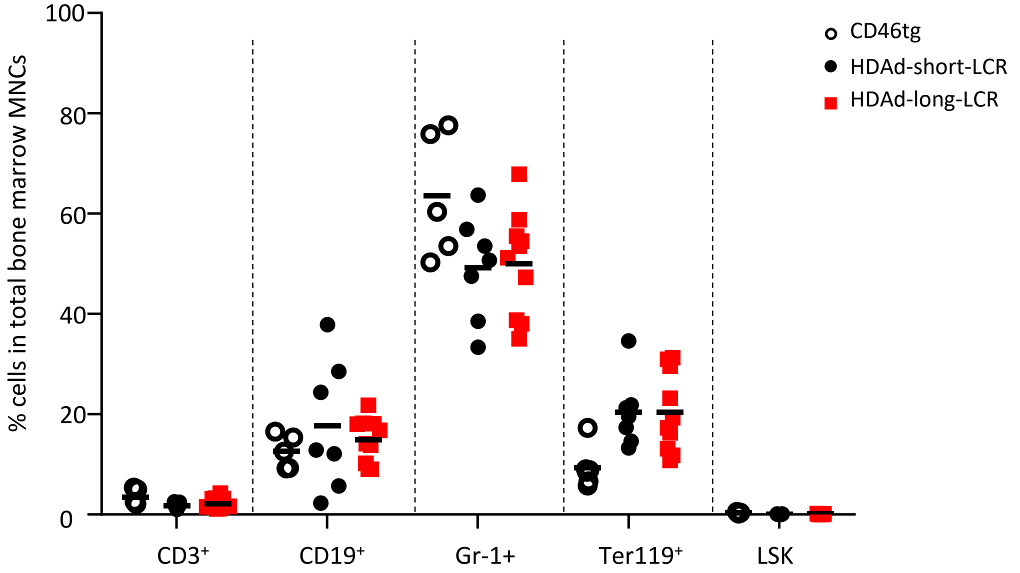

Figure S5. Cellular bone marrow composition of CD46 and treated Hbb^{th3}/CD46 mice at week 16 after *in vivo* transduction. The differences between the groups were not significant. Statistical analyses were performed using two-way ANOVA.

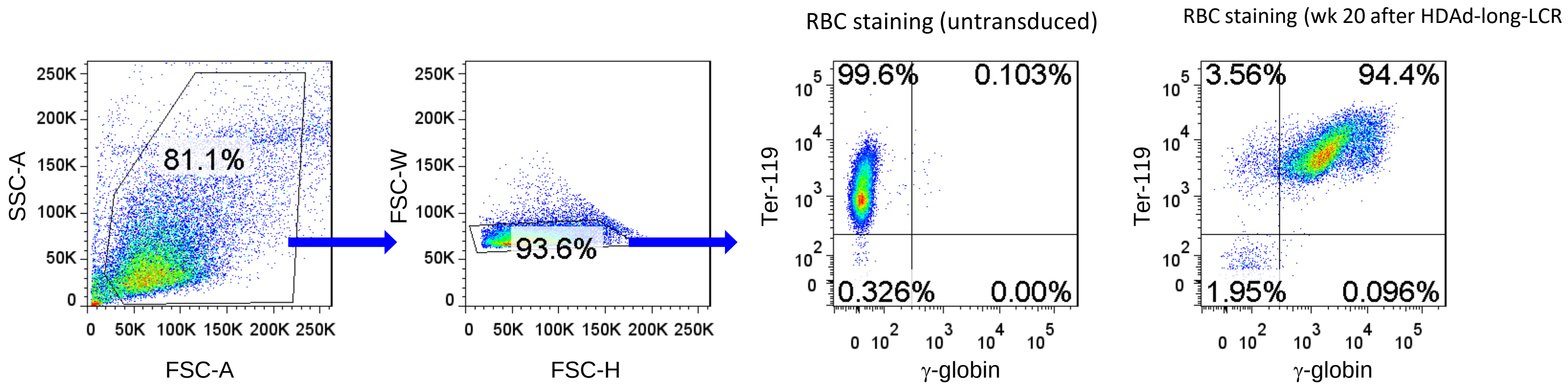

Figure S6. Human γ-globin gating strategy. Fixed and permeabilized RBCs from CD46/Hbb^{th3} mice were stained for the erythroid marker Ter-119 and intracellular γ-globin.

Name	Description	Fold Chang
HIST1H2BD	histone cluster 1, H2bd	-4.16894
HIST1H2BL	histone cluster 1, H2bl	-4.11243
HIST1H1D	histone cluster 1, H1d	-4.09723
HIST1H2AJ	histone cluster 1, H2aj	-4.08546
HIST1H4F	histone cluster 1, H4f	-3.95791
HIST1H3J	histone cluster 1, H3j	-3.9001
HIST1H2BM	histone cluster 1, H2bm	-3.85489
HIST1H1B	histone cluster 1, H1b	-3.8492
HIST1H2BK	histone cluster 1, H2bk	-3.83586
HIST1H3D	histone cluster 1, H3d	-3.82914
HIST1H4A	histone cluster 1, H4a	-3.80989
HIST1H2BC	histone cluster 1, H2bc	-3.79098
HIST1H4I	histone cluster 1, H4i	-3.74475
HIST1H2BJ	histone cluster 1, H2bj	-3.74226
HIST1H4C	histone cluster 1, H4c	-3.72763
HIST1H2BH	histone cluster 1, H2bh	-3.72637
HIST1H3B	histone cluster 1, H3b	-3.72064
HIST1H4E	histone cluster 1, H4e	-3.71633
HIST2H2AB	histone cluster 2, H2ab	-3.64594
HIST1H2BB	histone cluster 1, H2bb	-3.61441
HIST1H2BF	histone cluster 1, H2bf	-3.60268
HIST1H4H	histone cluster 1, H4h	-3.58982
HIST1H2AE	histone cluster 1, H2ae	-3.57346
HIST1H2BI	histone cluster 1, H2bi	-3.57232
FAM132B	family with sequence similarity 132, member B	-3.50956
HIST1H4D	histone cluster 1, H4d	-3.49129
HIST1H3H	histone cluster 1, H3h	-3.48005
HIST1H1C	histone cluster 1, H1c	-3.45638
HIST1H2BO	histone cluster 1, H2bo	-3.45056
DUSP5	dual specificity phosphatase 5	-3.44396
HIST3H2BB	histone cluster 3, H2bb	-3.40433
HIST1H2AL	histone cluster 1, H2al	-3.36177
HIST2H2BF	histone cluster 2, H2bf	-3.36165
IFIT1B	interferon-induced protein with tetratricopeptide repeats 1B	-3.34631
HIST1H4L	histone cluster 1, H4l	-3.32914
MIR3687-1	microRNA 3687-1	-3.32186
PSAT1	phosphoserine aminotransferase 1	-3.30346
HIST1H2AB	histone cluster 1, H2ab	-3.29777
HIST1H2AK	histone cluster 1, H2ak	-3.25212
HIST1H2AI	histone cluster 1, H2ai	-3.19867
HIST1H2BN	histone cluster 1, H2bn	-3.19558
CRHBP	corticotropin releasing hormone binding protein	-3.19481
OSM	oncostatin M	-3.18292
HIST1H3I	histone cluster 1, H3i	-3.15126
HIST1H3F	histone cluster 1, H3f	-3.13395
CISH	cytokine inducible SH2-containing protein	-3.11057

HIST1H2AH	histone cluster 1, H2ah	-3.09537
HIST1H2BG	histone cluster 1, H2bg	-3.05428
HIST1H3C	histone cluster 1, H3c	-3.00309
SOCS2	suppressor of cytokine signaling 2	-2.98632
CREB3L1	cAMP responsive element binding protein 3-like 1	-2.96358
HIST1H2AG	histone cluster 1, H2ag	-2.96245
TRIB3	tribbles pseudokinase 3	-2.94498
HIST2H2BE	histone cluster 2, H2be	-2.92837
HIST1H2AM	histone cluster 1, H2am	-2.91216
HIST1H2AC	histone cluster 1, H2ac	-2.91095
MIR3648-1	microRNA 3648-1	-2.90462
CCDC71L	coiled-coil domain containing 71-like	-2.89333
EHF	ets homologous factor	-2.88862
HIST1H4B	histone cluster 1, H4b	-2.88564
SIAH2	siah E3 ubiquitin protein ligase 2	-2.88172
FN1	fibronectin 1	-2.85148
HIST2H3D	histone cluster 2, H3d	-2.79615
ASNS	asparagine synthetase (glutamine-hydrolyzing)	-2.79514
LRRC8A	leucine rich repeat containing 8 family, member A	-2.78705
HIST1H3G	histone cluster 1, H3g	-2.74349
HES6	hes family bHLH transcription factor 6	-2.64125
HIST2H2AC	histone cluster 2, H2ac	-2.63357
TMCC2	transmembrane and coiled-coil domain family 2	-2.60575
SH3BGRL3	SH3 domain binding glutamate-rich protein like 3	-2.54527
PIM1	Pim-1 proto-oncogene, serine/threonine kinase	-2.43758
HBB	hemoglobin, beta	-2.42757
MTRNR2L2	MT-RNR2-like 2	2.56095
NFAT5	nuclear factor of activated T-cells 5, tonicity-responsive	2.56404
MON2	MON2 homolog, regulator of endosome-to-Golgi trafficking	2.58157
CAPRIN2	caprin family member 2	2.59071
REV3L	REV3-like, polymerase (DNA directed), zeta, catalytic subunit	2.6038
VPS13D	vacuolar protein sorting 13 homolog D (S. cerevisiae)	2.6132
RGS16	regulator of G-protein signaling 16	2.69566
MDM2	MDM2 proto-oncogene, E3 ubiquitin protein ligase	2.70157
ASCC3	activating signal cointegrator 1 complex subunit 3	2.75748
MYO18A	myosin XVIIIA	2.78425
CCNG1	cyclin G1	2.78848
USP37	ubiquitin specific peptidase 37	2.8107
DCUN1D4	DCN1, defective in cullin neddylation 1, domain containing 4	2.85642
FBXO22	F-box protein 22	2.87496
PRKX	protein kinase, X-linked	2.88002
CD70	CD70 molecule	2.88148
ABCA5	ATP-binding cassette, sub-family A (ABC1), member 5	2.88428
NIN	ninein (GSK3B interacting protein)	2.89654
NSF	N-ethylmaleimide-sensitive factor	2.91992
ACSBG1	acyl-CoA synthetase bubblegum family member 1	2.95294
CHD6	chromodomain helicase DNA binding protein 6	2.96857

SYDE2	synapse defective 1, Rho GTPase, homolog 2 (C. elegans)	2.99254
TNS3	tensin 3	2.99463
TRANK1	tetratricopeptide repeat and ankyrin repeat containing 1	3.00411
TP53INP1	tumor protein p53 inducible nuclear protein 1	3.00882
EPPK1	epiplakin 1	3.01097
MED12L	mediator complex subunit 12-like	3.01097
LATS2	large tumor suppressor kinase 2	3.02556
ABCA1	ATP-binding cassette, sub-family A (ABC1), member 1	3.02931
SYNE1	spectrin repeat containing, nuclear envelope 1	3.02931
LRMP	lymphoid-restricted membrane protein	3.03051
XPC	xeroderma pigmentosum, complementation group C	3.0529
CDIP1	cell death-inducing p53 target 1	3.05832
VWCE	von Willebrand factor C and EGF domains	3.06392
CUL9	cullin 9	3.07469
MYO1A	myosin IA	3.08357
NDRG1	N-myc downstream regulated 1	3.1014
KITLG	KIT ligand	3.10443
HMBOX1	homeobox containing 1	3.13136
PRKY	protein kinase, Y-linked, pseudogene	3.14467
PIK3R3	phosphoinositide-3-kinase, regulatory subunit 3 (gamma)	3.15065
REC8	REC8 meiotic recombination protein	3.16604
CCR7	chemokine (C-C motif) receptor 7	3.17081
MAN2B2	mannosidase, alpha, class 2B, member 2	3.17794
SPATA13	spermatogenesis associated 13	3.18769
CTSV	cathepsin V	3.18824
ANKRD20A	ankyrin repeat domain 20 family, member A9, pseudogene	3.20157
HSPA2	heat shock 70kDa protein 2	3.20555
C11orf21	chromosome 11 open reading frame 21	3.20921
MARCKSL1	MARCKS-like 1	3.21462
APAF1	apoptotic peptidase activating factor 1	3.22393
SYTL1	synaptotagmin-like 1	3.23369
ZNF850	zinc finger protein 850	3.23393
FAM212B	family with sequence similarity 212, member B	3.23914
CENPP	centromere protein P	3.25194
TMEM2	transmembrane protein 2	3.2569
DGKH	diacylglycerol kinase, eta	3.25961
PGF	placental growth factor	3.26334
AMOTL1	angiomotin like 1	3.28511
ASTN2	astrotactin 2	3.30026
GADD45B	growth arrest and DNA-damage-inducible, beta	3.33799
ACER2	alkaline ceramidase 2	3.34056
ZC3H8	zinc finger CCCH-type containing 8	3.38569
GYS1	glycogen synthase 1 (muscle)	3.39438
PHLDA3	pleckstrin homology-like domain, family A, member 3	3.41463
TRIM22	tripartite motif containing 22	3.43605
ZNF337	zinc finger protein 337	3.46335
RPL23AP53	ribosomal protein L23a pseudogene 53	3.47076

DDB2	damage-specific DNA binding protein 2, 48kDa	3.49739
CDKN1A	cyclin-dependent kinase inhibitor 1A (p21, Cip1)	3.5055
KIAA0754	KIAA0754	3.51706
RORA	RAR-related orphan receptor A	3.56588
FDXR	ferredoxin reductase	3.57076
STARD4	StAR-related lipid transfer (START) domain containing 4	3.57583
PKHD1L1	polycystic kidney and hepatic disease 1 (autosomal recessive)-like 1	3.57856
ISYNA1	inositol-3-phosphate synthase 1	3.5832
MIR612	microRNA 612	3.5932
SEMA4D	sema domain, immunoglobulin domain (Ig), transmembrane domain (TM) and short cytop	3.60445
CYP1B1	cytochrome P450, family 1, subfamily B, polypeptide 1	3.66908
EZH2	enhancer of zeste 2 polycomb repressive complex 2 subunit	3.68412
PLK3	polo-like kinase 3	3.69717
DGKA	diacylglycerol kinase, alpha 80kDa	3.7685
DRAM1	DNA-damage regulated autophagy modulator 1	3.78444
LINC01021	long intergenic non-protein coding RNA 1021	3.78549
ICA1	islet cell autoantigen 1, 69kDa	3.78798
CA3	carbonic anhydrase III	3.80712
CDC42EP3	CDC42 effector protein (Rho GTPase binding) 3	3.85706
PAK6	p21 protein (Cdc42/Rac)-activated kinase 6	3.85706
RFX7	regulatory factor X, 7	3.88098
ITGAM	integrin, alpha M (complement component 3 receptor 3 subunit)	3.92075
TRIM5	tripartite motif containing 5	3.92075
FAT1	FAT atypical cadherin 1	3.96717
APOBEC3H	apolipoprotein B mRNA editing enzyme, catalytic polypeptide-like 3H	4.01823
TTYH3	tweety family member 3	4.04147
NOTCH1	notch 1	4.07187
ZNF81	zinc finger protein 81	4.10434
ANKRD18B	ankyrin repeat domain 18B	4.12802
ANKRD18A	ankyrin repeat domain 18A	4.14161
NYNRIN	NYN domain and retroviral integrase containing	4.24822
EDA2R	ectodysplasin A2 receptor	4.37132
LRP1	low density lipoprotein receptor-related protein 1	4.41101
APOC1	apolipoprotein C-I	4.51489
CARNS1	carnosine synthase 1	4.67829

Log Fold Ch	p-Value	p-Adj	Average Lo	Cluster	HCD34C-d1	HCD34C-GI	Gene ID	Alias
-2.05968	0.004901	0.998465	10.0226		7.87556	10.8514	3017	H2B.1B, H2
-2.03999	0.005544	0.998465	9.3073		7.16897	10.136	8340	H2B/C, H2E
-2.03465	0.005275	0.998465	10.1661		8.067	10.9884	3007	H1.3, H1D,
-2.0305	0.00534	0.998465	10.1883		8.09708	11.0097	8331	H2A/E, H2A
-1.98474	0.006652	0.998465	9.288		7.24615	10.1038	8361	H4, H4/C, H
-1.96351	0.007728	0.998465	8.6086		6.56626	9.42609	8356	H3/J, H3FJ
-1.94669	0.007358	0.998465	9.5221		7.5543	10.327	8342	H2B/E, H2B
-1.94456	0.006652	0.998465	11.859		9.93478	12.6556	3009	H1, H1.5, H
-1.93955	0.007054	0.998465	10.5201		8.58906	11.3185	85236	H2B/S, H2B
-1.93702	0.00743	0.998465	9.7873		7.84399	10.5882	8351	H3/B, H3FB
-1.92975	0.009037	0.998465	8.2892		6.28204	9.10283	8359	H4FA
-1.92257	0.007433	0.998465	10.564		8.66128	11.358	8347	H2B.1, H2B
-1.90487	0.011115	0.998465	7.5999		5.56429	8.42076	8294	H4/M, H4FI
-1.90391	0.00857	0.998465	9.354		7.45097	10.1495	8970	H2B/R, H2E
-1.89826	0.008502	0.998465	9.6514		7.76752	10.4435	8364	H4/G, H4FC
-1.89777	0.008227	0.998465	10.1466		8.27598	10.936	8345	H2B/J, H2B
-1.89555	0.00793	0.998465	11.1362		9.28414	11.9218	8358	H3/L, H3FL
-1.89388	0.00849	0.998465	9.8538		7.98253	10.6436	8367	H4/J, H4FJ
-1.86629	0.010082	0.998465	8.9463		7.08743	9.73567	317772	H2AB
-1.85376	0.010534	0.998465	8.9097		7.06934	9.69619	3018	H2B.1, H2B
-1.84907	0.009714	0.998465	9.9616		8.16395	10.7393	8343	H2B/G, H2E
-1.84391	0.012498	0.998465	7.9787		6.09204	8.77575	8365	H4/H, H4FH
-1.83732	0.010608	0.998465	9.3253		7.52805	10.1038	3012	H2A.1, H2A
-1.83686	0.010799	0.998465	9.1622		7.35965	9.94189	8346	H2B/K, H2E
-1.81129	0.011503	0.998465	9.3323		7.57582	10.1038	151176	C1QTNF15,
-1.80376	0.011399	0.998465	9.7163		7.98253	10.4833	8360	H4/B, H4FB
-1.79911	0.011506	0.998465	9.7859		8.06091	10.5513	8357	H3/K, H3F1
-1.78926	0.010989	0.998465	11.5035		9.81686	12.2606	3006	H1.2, H1C,
-1.78683	0.011931	0.998465	9.8147		8.10893	10.5766	8348	H2B.2, H2B
-1.78407	0.02346	0.998465	5.9052		3.68501	6.76812	1847	DUSP, HVH
-1.76737	0.016068	0.998465	7.8951		6.12744	8.67311	128312	H2BB
-1.74922	0.01332	0.998465	9.9132		8.26542	10.6642	8332	H2A.I, H2A,
-1.74917	0.013297	0.998465	9.9414		8.29426	10.6922	440689	-
-1.74257	0.01564	0.998465	8.525		6.83962	9.28583	439996	IFIT1L, BA1
-1.73515	0.016379	0.998465	8.3649		6.68189	9.12588	8368	H4.K, H4/K,
-1.73199	0.02515	0.998465	6.3345		4.37639	7.15616	1.01E+08	MIR3687, H
-1.72398	0.026185	0.998465	6.2592		4.29571	7.08269	29968	EPIP, NLS2,
-1.72149	0.02011	0.998465	7.4633		5.72698	8.23826	8335	H2A/M, H2
-1.70138	0.02149	0.998465	7.434		5.72698	8.20402	8330	H2A/D, H2A
-1.67747	0.016265	0.998465	10.267		8.72815	10.996	8329	H2A/C, H2A
-1.67608	0.016529	0.998465	10.0554		8.51526	10.7849	8341	H2B/D, H2E
-1.67573	0.035378	0.998465	4.9214		2.30856	5.84933	1393	CRF-BP, CRI
-1.67035	0.034522	0.998465	5.633		3.55238	6.48415	5008	-
-1.65593	0.018503	0.998465	9.3599		7.83331	10.0875	8354	H3.F, H3/F,
-1.64798	0.018292	0.998465	9.81		8.30461	10.5324	8968	H3/I, H3FI
-1.63718	0.02649	0.998465	7.3426		5.72698	8.09615	1154	BACTS2, CIS

-1.63011	0.021292	0.998465	8.7661		7.25684	9.49142	85235	H2A/S, H2A
-1.61083	0.021084	0.998465	9.4222		7.9598	10.136	8339	H2B.1A, H2
-1.58645	0.021229	0.998465	10.469		9.05668	11.1701	8352	H3.1, H3/C,
-1.57837	0.038401	0.998465	6.4387		4.78385	7.20891	8835	CIS2, CISH2
-1.56734	0.038517	0.998465	6.5643		4.95124	7.32496	90993	OASIS
-1.56679	0.022725	0.998465	10.2801		8.89149	10.9758	8969	H2A.1B, H2
-1.55826	0.031495	0.998465	7.6221		6.15057	8.34423	57761	C20ORF97,
-1.5501	0.023619	0.998465	10.5241		9.16001	11.2138	8349	GL105, H2E
-1.54209	0.025136	0.998465	9.8144		8.45151	10.5046	8336	H2A.1, H2A
-1.54149	0.026071	0.998465	9.3567		7.98575	10.0494	8334	H2A/L, H2A
-1.53835	0.038649	0.998465	6.9146		5.40031	7.65145	1.01E+08	MIR3648, H
-1.53273	0.029291	0.998465	8.5249		7.14038	9.2231	168455	C7ORF74
-1.53038	0.048743	0.998465	4.2596		1.09064	5.25469	26298	ESE3, ESE3I
-1.52889	0.02868	0.998465	8.7833		7.41323	9.47716	8366	H4/I, H4FI
-1.52693	0.025056	0.998465	10.7814		9.44945	11.463	6478	HSIAH2
-1.51171	0.045348	0.998465	6.5321		5.00299	7.27636	2335	CIG, ED-B, F
-1.48344	0.038848	0.998465	7.6468		6.28204	8.34423	653604	-
-1.48292	0.037699	0.998465	7.821		6.46843	8.51453	440	ASNSD, TS1
-1.47874	0.030573	0.998465	9.6879		8.40418	10.3585	56262	AGM5, LRR
-1.45601	0.035765	0.998465	8.7097		7.43222	9.38084	8355	H3/H, H3FH
-1.40122	0.041735	0.998465	8.736		7.52805	9.38848	55502	C-HAIRY1, H
-1.39702	0.039539	0.998465	9.4259		8.23869	10.071	8338	H2A, H2A-G
-1.3817	0.037188	0.998465	12.3089		11.1613	12.9403	9911	HUCEP11
-1.34782	0.044933	0.998465	9.6141		8.48828	10.241	83442	HEL-S-297,
-1.28545	0.049839	0.998465	11.4782		10.4379	12.0771	5292	PIM
-1.27951	0.049193	0.998465	18.094		17.0653	18.6884	3043	CD113T-C,
1.35668	0.048418	0.998465	8.6076		9.24707	7.44708	1E+08	HN2, MTRN
1.35842	0.046305	0.998465	8.9605		9.59669	7.80782	10725	NF-AT5, NF
1.36825	0.046948	0.998465	8.5926		9.2363	7.41751	23041	-
1.37335	0.046079	0.998465	8.6273		9.27233	7.44708	65981	C1QDC1, EI
1.38062	0.04525	0.998465	8.6073		9.2551	7.41751	5980	POLZ, REV3
1.38582	0.043384	0.998465	8.8405		9.4874	7.65145	55187	-
1.43064	0.042557	0.998465	8.045		8.7185	6.76812	6004	A28-RGS14
1.4338	0.033081	0.998465	10.7742		11.4259	9.55732	4193	ACTFS, HDM
1.46335	0.033522	0.998465	9.1731		9.84283	7.89624	10973	ASC1P200,
1.47729	0.044477	0.998465	7.124		7.83688	5.70989	399687	MYSPDZ, SI
1.47948	0.032051	0.998465	9.1507		9.82587	7.85271	900	CCNG
1.49093	0.04442	0.998465	6.9552		7.67883	5.50017	57695	-
1.51421	0.039691	0.998465	7.1728		7.89623	5.70989	23142	-
1.52354	0.039729	0.998465	7.0478		7.77869	5.55553	26263	FBX22, FIST
1.52608	0.042353	0.998465	6.7293		7.47407	5.18619	5613	PKX1
1.52681	0.048039	0.998465	6.1452		6.92257	4.4754	970	CD27L, CD2
1.52821	0.045365	0.998465	6.3952		7.1576	4.7843	23461	ABC13, EST
1.53433	0.037639	0.998465	7.1554		7.88593	5.66025	51199	SCKL7
1.54593	0.042245	0.998465	6.494		7.25684	4.87414	4905	SKD2
1.56215	0.042289	0.998465	6.2831		7.06326	4.58588	23205	BG, BG1, BG
1.56977	0.034008	0.998465	7.1579		7.89965	5.60884	84181	CHD-6, CHD

1.58137	0.044929	0.998465	4.6044	5.58141	1.4701	84144	-
1.58238	0.046359	0.998465	5.4016	6.26094	3.30042	64759	TEM6, TENS
1.58694	0.023187	0.998465	9.2228	9.93145	7.78484	9881	LBA1
1.5892	0.021306	0.998465	10.2802	10.9832	8.86061	94241	SIP, TP53DI
1.59023	0.043808	0.998465	4.6211	5.59833	1.4701	83481	EPIPL, EPIP
1.59023	0.043808	0.998465	4.6211	5.59833	1.4701	116931	NOPAR, TN
1.5972	0.044904	0.998465	5.2585	6.13905	3.01465	26524	KPM
1.59899	0.042724	0.998465	4.6376	5.61505	1.4701	19	ABC-1, ABC
1.59899	0.042724	0.998465	4.6376	5.61505	1.4701	23345	8B, ARCA1,
1.59956	0.044817	0.998465	5.0756	5.98031	2.65792	4033	JAW1
1.61018	0.023169	0.998465	8.6298	9.3518	7.13814	7508	RAD4, XP3,
1.61274	0.03825	0.998465	6.0789	6.88859	4.22528	29965	C16ORF5, C
1.61538	0.039549	0.998465	5.8629	6.68981	3.92251	220001	URG11, VW
1.62044	0.031438	0.998465	6.8613	7.6303	5.18619	23113	H7AP1, PAI
1.6246	0.039678	0.998465	4.6858	5.66409	1.4701	4640	BBMI, DFN
1.63292	0.038727	0.998465	4.7016	5.68007	1.4701	10397	CAP43, CM
1.63433	0.040367	0.998465	5.1348	6.04345	2.65792	4254	FPH2, FPHH
1.64679	0.035815	0.998465	5.9111	6.74403	3.92251	79618	HNF1LA, H(
1.65291	0.031127	0.998465	6.4973	7.29367	4.6885	5616	PRKXP3, PR
1.65565	0.033365	0.998465	6.1437	6.96232	4.22528	8503	P55, P55-G
1.66268	0.028663	0.998465	6.7316	7.51919	4.9587	9985	HR21SPB, R
1.66485	0.035252	0.998465	4.7629	5.74228	1.4701	1236	BLR2, CC-CI
1.66809	0.035582	0.998465	5.5394	6.41213	3.30042	23324	-
1.67251	0.03299	0.998465	5.9512	6.78894	3.92251	221178	ARHGEF29,
1.67276	0.034433	0.998465	4.7778	5.75743	1.4701	1515	CATL2, CTS
1.67878	0.0344	0.998465	5.5568	6.43114	3.30042	284232	-
1.68057	0.03364	0.998465	4.7926	5.77241	1.4701	3306	HSP70-2, H
1.68222	0.029388	0.998465	6.3737	7.18586	4.4754	29125	-
1.68465	0.033118	0.998465	5.7136	6.57484	3.53884	65108	F52, MACM
1.68882	0.033946	0.998465	5.0248	5.96735	2.18295	317	APAF-1, CEI
1.69318	0.033677	0.998465	5.2356	6.15057	2.65792	84958	JFC1, SLP1
1.69329	0.030849	0.998465	5.9838	6.82532	3.92251	342892	ZNF850P
1.69561	0.033245	0.998465	5.0372	5.98031	2.18295	55924	C1ORF183
1.7013	0.02297	0.998465	7.1989	7.97931	5.44261	401541	CENP-P
1.7035	0.023981	0.998465	6.9823	7.77125	5.18619	23670	-, CEMIP2
1.7047	0.025814	0.998465	6.6517	7.45562	4.7843	160851	DGKETA
1.70635	0.020559	0.998465	7.6426	8.41138	5.93532	5228	D12S1900,
1.71594	0.031216	0.998465	5.074	6.01852	2.18295	154810	JEAP
1.72258	0.030577	0.998465	5.086	6.03104	2.18295	23245	BA67K19.1
1.73898	0.028174	0.998465	4.9057	5.887	1.4701	4616	GADD45BE
1.74009	0.027675	0.998465	5.8039	6.67393	3.53884	340485	ALKCDASE2
1.75945	0.024815	0.998465	6.0893	6.94258	3.92251	84524	FLIZ1, ZC3H
1.76315	0.024511	0.998465	6.0952	6.94919	3.92251	2997	GSY, GYS
1.77173	0.022283	0.998465	6.4214	7.25684	4.35576	23612	TIH1
1.78075	0.025467	0.998465	5.3893	6.31312	2.65792	10346	GPSTAF50,
1.79217	0.021247	0.998465	6.3575	7.20255	4.22528	26152	-
1.79525	0.021026	0.998465	6.3625	7.20808	4.22528	644128	RPL23A_20

1.80628	0.012289	0.998465	8.9264	9.70121	7.15616	1643	DDBB, UV-[illegible]
1.80962	0.011495	0.998465	9.4554	10.2263	7.7014	1026	CAP20, CD[illegible]
1.81437	0.022811	0.998465	5.2556	6.20681	2.18295	643314	-
1.83426	0.014368	0.998465	7.499	8.30718	5.55553	6095	NR1F1, RO[illegible]
1.83623	0.011387	0.998465	8.7901	9.57442	6.96496	2232	ADXR
1.83828	0.014823	0.998465	7.3025	8.11776	5.32009	134429	-
1.83938	0.018924	0.998465	6.2207	7.08743	3.92251	93035	PKHDL1
1.84125	0.020397	0.998465	5.1082	6.09204	1.4701	51477	INO1, INOS
1.84527	0.01771	0.998465	6.4443	7.29885	4.22528	693197	MIRN612, [illegible]
1.84978	0.019983	0.998465	5.688	6.60026	3.01465	10507	C9ORF164,
1.87542	0.012118	0.998465	7.6993	8.51303	5.70989	1545	CP1B, CYPI[illegible]
1.88132	0.009869	0.998465	8.8007	9.59669	6.90228	2146	ENX-1, ENX
1.88642	0.01607	0.998465	6.3004	7.17462	3.92251	1263	CNK, FNK, [illegible]
1.91399	0.014239	0.998465	6.4577	7.32957	4.08182	1606	DAGK, DAG
1.92008	0.013325	0.998465	6.6657	7.52805	4.35576	55332	DRAM
1.92048	0.010273	0.998465	7.8144	8.63693	5.75788	643401	-
1.92143	0.015864	0.998465	5.6468	6.58336	2.65792	3382	ICA69, ICA[illegible]
1.9287	0.015215	0.998465	5.8308	6.75161	3.01465	761	CAIII, CAR3
1.9475	0.014332	0.998465	5.327	6.31312	1.4701	10602	BORG2, CE[illegible]
1.9475	0.014332	0.998465	5.327	6.31312	1.4701	56924	PAK5
1.95642	0.008562	0.998465	8.1732	8.99733	6.0933	64864	RFXDC2
1.97113	0.012489	0.998465	6.2028	7.1053	3.53884	3684	CD11B, CR[illegible]
1.97113	0.008529	0.998465	7.9394	8.77168	5.80433	85363	RNF88, TRI[illegible]
1.98811	0.010019	0.998465	6.9565	7.82255	4.58588	2195	CDHF7, CD[illegible]
2.00656	0.011689	0.998465	5.4533	6.44055	1.4701	164668	A3H, ARP-1
2.01488	0.011353	0.998465	5.4718	6.4592	1.4701	80727	-
2.02569	0.011058	0.998465	5.6699	6.63347	2.18295	4851	AOS5, AOV
2.03715	0.009111	0.998465	6.6766	7.56725	4.08182	347344	HFZ20, MR[illegible]
2.04545	0.006595	0.998465	8.0262	8.87422	5.75788	441459	BA255A11.
2.05019	0.009352	0.998465	6.3499	7.26216	3.53884	253650	-
2.08686	0.007568	0.998465	6.7692	7.66685	4.08182	57523	CGIN1, KIA[illegible]
2.12807	0.005508	0.998465	7.567	8.44451	5.03858	60401	EDA-A2R, E
2.14111	0.006982	0.998465	6.2449	7.18586	3.01465	4035	A2MR, APC
2.17469	0.006366	0.998465	5.9914	6.96232	2.18295	341	APO-CI, AP[illegible]
2.22598	0.004434	0.998465	7.0438	7.9598	4.08182	57571	ATPGD1

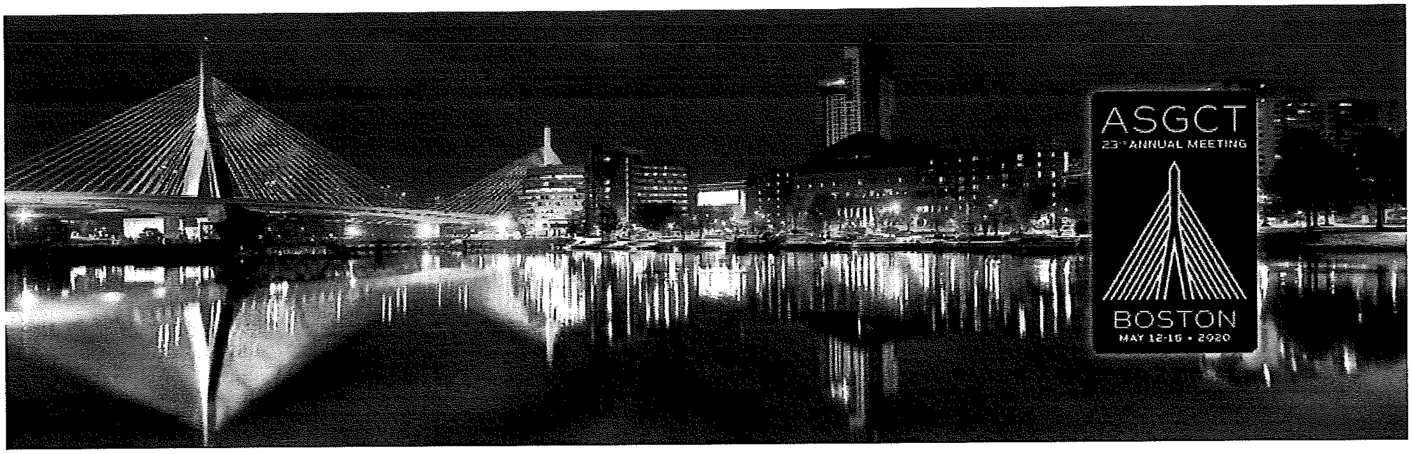

Print this Page for Your Records   Close Window

Control/Tracking Number: 2020-A-1700-ASGCT
Activity: Abstract
Current Date/Time: 1/30/2020 11:53:38 AM

***In vivo* HSC gene therapy for hemoglobinopathies: a proof of concept evaluation in rhesus macaques**

Author Block: Chang Li[1], Hongjie Wang[1], Audrey Germond[2], Alan Ung[2], Kate Guerriero[2], Jason Ogle[2], Veronica Nelson[3], Dean Jeffrey[2], Cari Coles[4], Aphrodite Georgakopoulou[1], Evangelia Yannaki[5], Thalia Papayannopoulou[1], Zsuzsanna Izsvak[6], Hans-Peter Kiem[3], Andre Lieber[7]

[1]Medicine, University of Washington, Seattle, WA,[2]WaNPRC, University of Washington, Seattle, WA,[3]FHCRC, Seattle, WA,[4]University of Washington, University of Washington, Seattle, WA,[5]Cell and Gene Therapy Center, Thessaloniki, Greece,[6]MDC, Berlin, Germany,[7]Medicine, University of Washington, SEATTLE, WA

Abstract:
Current gene therapy or genome editing studies for hemoglobinopathies require highly sophisticated medical facilities to perform hematopoietic stem cell (HSC) collections/selections and genetic modifications. In addition, patients receive high-dose chemotherapy to facilitate engraftment of gene-modified cells. Thus, current gene therapy protocols will not be accessible to most patients suffering from hemoglobinopathies. Here we describe a highly portable and scalable approach using *in vivo* HSC gene therapy to potentially overcome these limitations. The central idea of our *in vivo* HSC gene therapy approach is to mobilize HSCs from the bone marrow, and while they circulate at high numbers in the periphery, transduce them with an intravenously injected HSC-tropic, helper-dependent adenovirus HDAd5/35++ gene transfer vector system. Transduced cells return to the bone marrow where they persist long-term. Transgene integration is either achieved by a *Sleeping Beauty* transposase (SB100x) in a random pattern or by homology-directed-repair into a safe genomic harbor site. Currently, an *in vivo* selection system (involving the $mgmt^{P140K}$ gene/low-dose O^6BG/BCNU) is employed to achieve 80-100% marking levels in peripheral blood cells. We demonstrated safety and efficacy of our approach in mouse models for thalassemia intermedia and hemophilia A, where we achieved a phenotypic correction. We now present data in rhesus macaques. The first animal was followed for 22 weeks after *in vivo* HSC transduction with a human-γ-globin expressing HDAd5/35++ vector using SB100x for integration. Treatment with G-CSF/AMD3100 resulted in efficient HSC mobilization. Intravenous injection of the HDAd5/35++ vector system (total $1.5x10^{12}$ vp/kg, in two doses) into mobilized animals was well tolerated after pretreatment with steroids and the IL-6 receptor inhibitor, tocilizumab. After *in vivo* selection, gamma-globin marking in peripheral red blood cells rose to ~90% and was stable during the study duration (see Figure). Gamma-globin levels in red blood cells were ~18% of adult alpha1-globin (by HPLC). There were no histological abnormalities at necropsy (week 22). A second mobilized animal was injected with an HDAd5/35++ vector system that allowed for targeted integration into the AAVS1 locus (total $3.2x10^{12}$ vp/kg in two doses). This animal was pre-treated with the IL-1beta receptor antagonist Anakinra, in addition to steroids and tocilizumab. Despite the relatively high vector dose, no side effects of the intravenous HDAd5/35++ injection were observed. A third animal will be started in February. Our data suggest that acute responses to the vector injection can be prevented with appropriate pretreatment. This is the first proof-of-concept study that *in vivo* HSC gene therapy could be feasible in humans and provide the necessary portability and accessibility to reach patients in places with limited medical resources. Ongoing studies involve the optimization of HSC mobilization, gene transfer vectors, and *in vivo* selection.

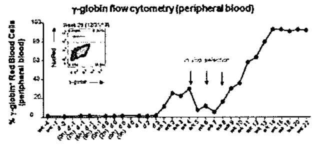

:

Author Disclosure Information:

C. Li: None.

Presenter Information (Complete):
- **Membership Status**: Associate Member
- **Gender:** Male
- **United States Citizenship status:** Not a US Citizen
- **If you consider yourself a member of one of the following U.S. under-represented groups, please indicate which one below:** N/A

Category (Complete): Hematologic & Immunologic Diseases ; Gene Targeting & Gene Correction (basic development of novel technologies for genome editing, with/without site-specific endonuclease)*

Presentation Preference (Complete): Either Oral or Poster

Questionnaire (Complete):
- **Clinical Trial/Clinical Study**: NO, this abstract is NOT a Clinical Trial/Clinical Study
- **Prior Publication**: No, my abstract has NOT been previously published
- **I confirm that all authors on this abstract approve this submission.** : True
- **Social Media Consideration**: Yes
- **Disease Focus of Abstract**: Hemoglobinopathies
- **National Heart, Lung and Blood Institute (NHLBI)** : True
- **Other Foundation Funding** : Bill and Melinda Gates Foundation

Awards (Complete):
- **Trainee Travel Award Consideration**: I am applying as a postdoctoral fellow
- **Underrepresented Minority Travel Award Consideration**: I am not applying
- **Under-Represented Minority group**: N/A

Keywords (Complete): Hematologic Diseases ; Hematopoietic Stem Cells ; Animal Models

Status: Complete

To become a member of the American Society of Gene & Cell Therapy go to http://www.asgct.org/membership/join-asgct.

Questions about the **ASGCT Annual Meeting**? Contact ASGCT at info@asgct.org.

Questions? Contact cOASIS Support at asgct@support.ctimeetingtech.com.
Feedback

Powered by cOASIS, The Online Abstract Submission and Invitation System SM
© 1996 - 2020 CTI Meeting Technology All rights reserved. Privacy Policy
